# Studies of Water Films and Carbonation via Neutron Scattering and Infrared Adsorption: In Situ Studies of Mg(OH)_2_ and Ca(OH)_2_

**DOI:** 10.1021/acs.jpcc.6c00841

**Published:** 2026-03-24

**Authors:** Hubert King, Ryan Murphy, Avery Baumann, Robert Dalgliesh, Dirk Honecker, Greg Smith

**Affiliations:** † NIST Center for Neutron Research, 10833 National Institute of Standards and Technology, 100 Bureau Dr, Gaithersburg, Maryland 20878, United States; ‡ Material Measurement Laboratory, National Institute of Standards and Technology, 100 Bureau Dr., Gaithersburg, Maryland 20878, United States; § 120797 ISIS Pulsed Neutron and Muon Source, STFC Rutherford Appleton Laboratory, Harwell Campus, Didcot OX11 0QX, U.K.; ∥ Department of Chemical & Biomolecular Engineering, Center for Neutron Science, University of Delaware, Newark, Delaware 19716, United States

## Abstract

Small-angle neutron scattering (SANS) and polarization-modulation infrared reflection–absorption spectroscopy (PM-IRRAS) were used to investigate the coupled evolution of nanometer water films and carbonation products in Ca­(OH)_2_ and Mg­(OH)_2_ under humidified CO_2_. Quantitative SANS modeling demonstrates that subnanometer adsorbed films on Ca­(OH)_2_ and thicker (≈1–2 nm) D_2_O films on Mg­(OH)_2_ mediate ion transport and isotopic exchange at buried interfaces. Infrared spectra confirm H–D exchange on Mg­(OH)_2_ but not on Ca­(OH)_2_, revealing distinct surface accessibility. In situ, both hydroxides initially form amorphous hydrated carbonates—ACC and AMC—but diverge as Ca­(OH)_2_ spontaneously dehydrates to CaCO_3_ at room temperature while Mg­(OH)_2_ remains hydrated. This divergence reflects the higher hydration affinity of Mg^2+^ and links isotope-dependent water-film stability to the persistence of amorphous phases. Together, these findings show that interfacial water films dictate whether carbonation proceeds by porosity generation (Calcium) or densification (Magnesium), providing mechanistic insight into mineral carbonation, cement durability, and low-temperature CO_2_ alteration processes.

## Graphical abstract

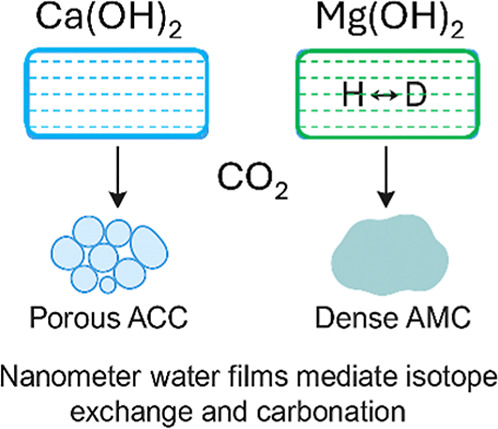

## Introduction

Over the past decades, numerous studies have examined the effects of atmospheric carbon dioxide on mineral and cementitious materials. Reports from the National Academy of Sciences^1^ and related assessments^2–4^ outline reactive pathways in which CO_2_ is converted directly into stable mineral forms. Among these, reactions of alkaline-earth oxides and hydroxides—especially those of calcium and magnesium—have attracted attention due to their natural abundance, industrial availability, and cyclic reactivity.^5^ Their relevance extends to the cement industry, where portlandite and calcium–silicate–hydrate phases are known to react with atmospheric CO_2_, altering microstructure and durability.^6,7^ Carbonation also plays a role in the weathering of rocks and man-made structures, as atmospheric CO_2_ dissolves in surface moisture to form weak carbonic acid that gradually carbonates alkaline materials and lowers local alkalinity over time. Materials such as nanolimes have been developed to mitigate or reverse such degradation processes.^8^ Together, these considerations motivate a detailed examination of carbonation mechanisms in calcium- and magnesium-based hydroxide systems.

Previous studies have established that carbonation of hydroxide and oxide surfaces proceeds only in the presence of an adsorbed water layer. Below a critical relative humidity, CO_2_ uptake is negligible, whereas at higher humidity thin, quasi-liquid water films promote ion mobility and carbonate formation.^9–15^ Beyond this kinetic binary role, theory also predicts distinct reaction pathways for calcium and magnesium hydroxides: thermodynamic analyses and density functional theory calculations indicate that Ca­(OH)_2_ readily converts to stable CaCO_3_, whereas Mg­(OH)_2_ preferentially forms hydrated carbonate intermediates (e.g., nesquehonite and hydromagnesite) that can hinder complete transformation at low temperature.^16,17^ Together, these results suggest that interfacial hydration may not only enable carbonation but also influence whether hydrated amorphous or crystalline intermediates persist.

Despite this consensus, key properties of the interfacial films themselves, including their thickness, structure, and isotopic dependence under reaction conditions are inferred, particularly at buried solid–gas interfaces within powder samples. Here, we combine small-angle neutron scattering (SANS) and polarization-modulation infrared reflection–absorption spectroscopy (PM-IRRAS) to probe interfacial water films in situ during carbonation under both H_2_O- and D_2_O-humidified CO_2_. Neutron contrast between hydrogenous and deuterated systems enables quantitative determination of film thickness, density, and hydrogen–deuterium exchange at reactive interfaces, providing direct structural insight into the role of interfacial water during carbonation.

Oomi identified a critical reactivity threshold of approximately 2.1–2.4 monolayers of adsorbed water for olivine carbonation, above which rapid reaction occurs.^13^ This threshold highlights that interfacial water must reach a minimum thickness and continuity to support ion transport and carbonate formation, an insight directly relevant to hydroxide carbonation. Isotopic effects further underscore the sensitivity of these processes to water structure and dynamics. In cementitious systems, hydration and carbonation reactions are known to proceed more slowly in D_2_O than in H_2_O, reflecting altered hydrogen-bonding networks and reduced diffusivity in heavy water.^18^ By examining Ca­(OH)_2_ and Mg­(OH)_2_, key phases also present in hydrated cement, under controlled H_2_O- and D_2_O-humidified conditions, the present work leverages isotopic contrast to probe how water-film structure, stability, and dynamics regulate carbonation pathways and product morphology.

Despite extensive study of hydroxide carbonation, the physical mechanisms controlling divergent reaction pathways in calcium and magnesium systems remain incompletely resolved. In particular, the role of nanometer-scale interfacial water films (known to mediate ion transport and carbonate formation) has largely been inferred rather than directly quantified. Here, we test the hypothesis that divergent carbonation pathways in calcium and magnesium hydroxides are governed by the thermodynamic stability and chemical accessibility of nanometer-scale interfacial water films. Because Ca^2+^ and Mg^2+^ differ markedly in hydration energetics, their associated water films are expected to exhibit distinct thicknesses, isotopic responses, and abilities to stabilize hydrated amorphous carbonate intermediates. By combining in situ neutron scattering with surface-sensitive infrared spectroscopy under controlled H_2_O and D_2_O humidification, we directly probe interfacial water structure and link it to carbonation products and nanoscale structural evolution.

## Materials and Methods

### Materials

High-purity powders of Ca­(OH)_2_ (American Elements, 98%) and Mg­(OH)_2_ (US Nano, brucite phase) were used as received.^1^ The Ca­(OH)_2_ consisted primarily of portlandite with minor CaCO_3_ contamination, while Mg­(OH)_2_ showed no detectable impurities. BET surface areas were 1.7 m^2^ g^–1^ and 18.1 m^2^ g^–1^, respectively. Each powder was stored in a desiccator prior to use. Three representative samples—CH1 (Ca­(OH)_2_), MH1and MH4 (Mg­(OH)_2_)—were examined under varying humidified gas environments (see Table S1). MH1 and MH4 differ only in their treatment history, as documented in that table.[Fn fn1]

### Gas Exposure and Humidification

CO_2_ and N_2_ (ultrahigh purity) were used as carrier gases. Humidified streams were generated by bubbling through high-purity H_2_O or D_2_O reservoirs and combined via mass-flow controllers to achieve target RH levels. For D_2_O, measured RH values were corrected by a factor of 1.055 (footnote Table S1) to account for isotopic calibration differences. Experiments were conducted at 25–30 °C and 70–98% RH. See Table S1 for a summary of conditions used.

### Neutron Scattering

Experiments were performed at the LARMOR instrument, ISIS Neutron and Muon Source (UK), using simultaneous small- and wide-angle neutron scattering (SANS/WANS) geometry. Powder samples (∼1 mm thick, 20 mm diameter) were loaded into titanium cells integrated with a custom gas-handling manifold that allowed controlled flow of humidified gases across the sample (Figure 1). The incident wavelength range was 0.9–13.5 Å, providing a combined *q* range of 0.005–8.4 Å^–1^. Data reduction (Mantid^19^) included dark-current correction, normalization to transmitted intensity, absolute calibration using a polymer standard, and background subtraction.

**Figure 1:**
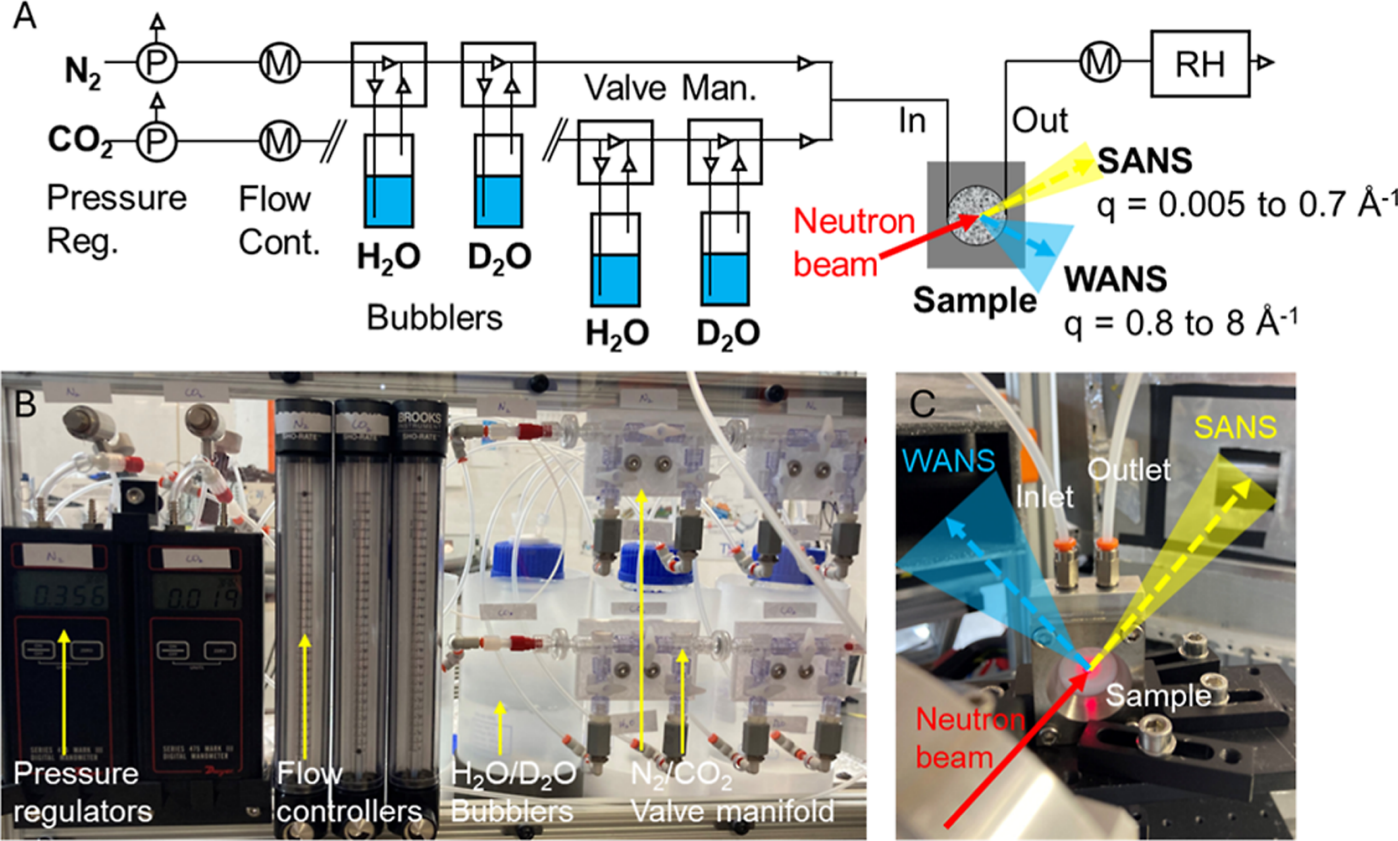
Schematic of (A) the gas-handling system and (B) neutron scattering geometry for powdered samples on the beamline. Relative humidity and temperature were continuously monitored using a Thorlabs TSP01 sensor (RH). Temperature was maintained at room temperature by the instrument hutch environment. Samples were exposed to either dry or humidified gas streams (H_2_O or D_2_O), generated upstream and delivered under steady-flow conditions. The reported temperature and relative humidity values (Table S1) correspond to steady-state conditions during neutron measurements.

The SANS regime (0.005–0.7 Å^–1^) resolved nano- and mesoscale structural changes, while WANS (0.8–8.3 Å^–1^) monitored crystalline-phase evolution. The absolute intensity calibration allowed quantitative analysis of Porod scattering incoherent background, providing sensitivity to hydrogen/deuterium content and thus water-film growth and isotopic exchange. Detailed sample conditions, exposure times, and fitted parameters are summarized in Supporting Information.

Unlike X-ray scattering, which is dominated by electron density, neutron scattering is uniquely sensitive to atomic nuclei and exhibits exceptionally strong incoherent scattering from hydrogen. In practice, this means that even small changes in hydrogen content within the illuminated sample volume produce measurable changes in the q-independent background intensity. In this respect, the approach is conceptually analogous to quantitative ^1^H NMR, where integrated signal intensity directly reports hydrogen content. Here, we exploit this nuclear sensitivity to use the high-q incoherent background as a direct, in situ reporter of adsorbed water films and hydrogen–deuterium exchange at buried interfaces. Because D_2_O exhibits dramatically reduced incoherent scattering relative to H_2_O, isotopic substitution provides an internal contrast switch that allows quantitative determination of film growth and exchange processes under reaction conditions.

### Infrared Spectroscopy (PM-IRRAS)

Complementary chemical and isotopic information were obtained via polarization modulation infrared reflection–absorption spectroscopy (PM-IRRAS) using a Thermo Nicolet iS50 spectrometer equipped with a J.A. Woollam cell.

Polarization-modulation infrared reflection–absorption spectroscopy (PM-IRRAS) is a surface-sensitive infrared method that enhances vibrational signals from molecules adsorbed at a solid surface while minimizing contributions from the gas phase and bulk material. By rapidly modulating the polarization of the incident infrared beam, PM-IRRAS isolates absorption features associated with surface hydroxyls, adsorbed water, and reaction products. In this study, PM-IRRAS is used to monitor H–D exchange and carbonate formation at hydroxide surfaces under controlled gas exposure.

Ca­(OH)_2_ and Mg­(OH)_2_ powders (3 wt %) were dispersed in isopropanol, spin-coated onto gold substrates, and exposed to the same sequential gas environments used in the neutron experiments: D_2_O-humidified N_2_ (18–24 h), D_2_O-humidified CO_2_ (18–24 h), and dry N_2_ (∼3 h). Gas composition and humidity were controlled by dual mass-flow controllers (Alicat Scientific) delivering dry and humidified CO_2_ streams through a bubbler at room temperature. Relative humidity for D_2_O experiments (≈65–70%) was estimated from prior calibrations, as the LI-850 humidity sensor (LI-COR Environmental) detects only H_2_O. Spectra were collected every 2 min at 30 °C using OMNIC software. The PM-IRRAS technique isolates vibrational modes of surface species and adsorbed films, enabling identification of O–H and O–D stretching vibrations, carbonate bands, and broad absorptions associated with liquid-like D_2_O films, Figure 2. The results presented are the raw data. There is no baseline correction/subtraction performed, and the Bessel function is not subtracted from the data.

**Figure 2:**
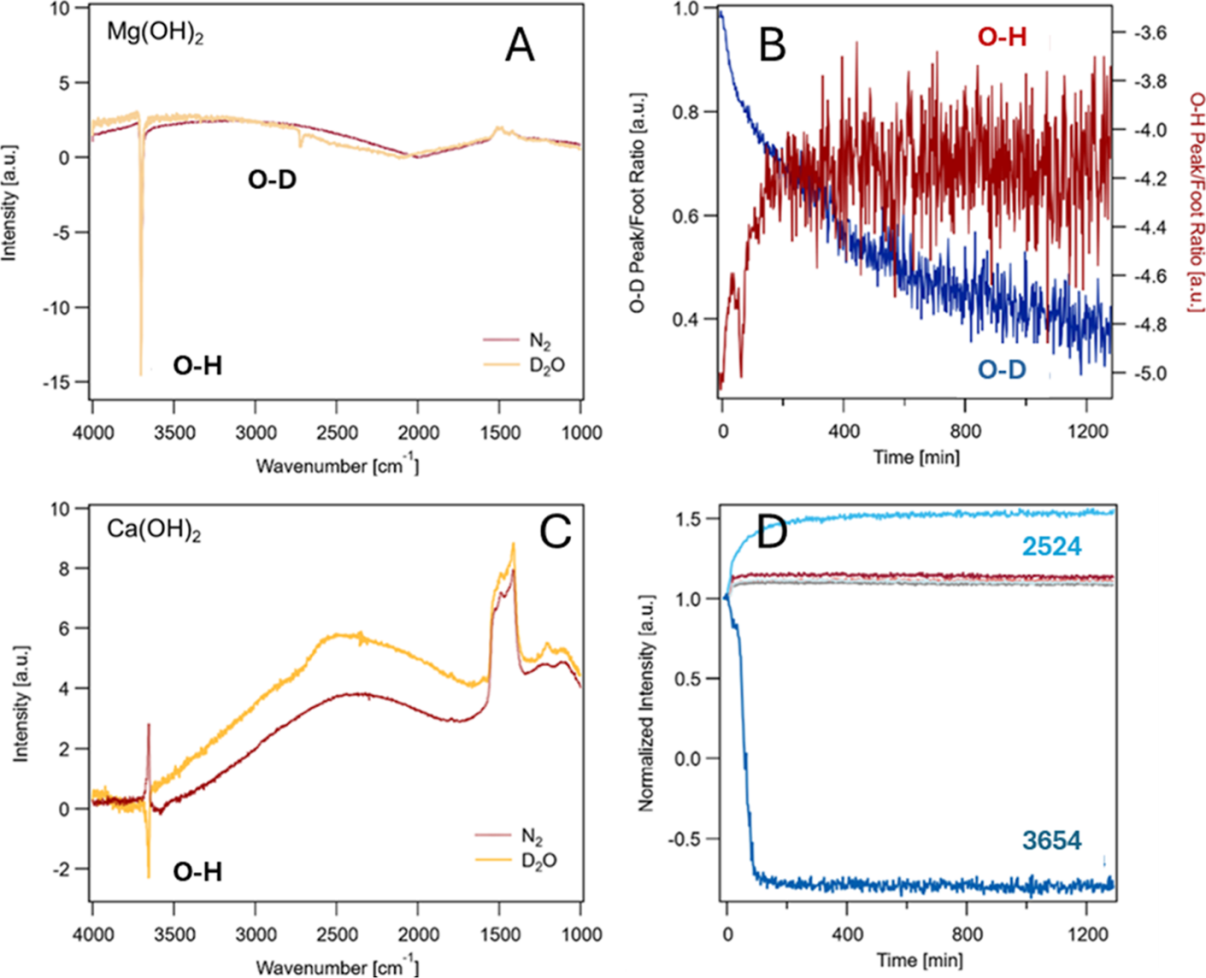
PM-IRRAS spectra and temporal evolution of hydroxide samples under N_2_ and D_2_O-humidified N_2_. (A) Mg­(OH)_2_ spectra showing negative-intensity hydroxide (O–H, ≈3700 cm^–1^) and deuteroxide (O–D, ≈2512 cm^–1^) stretching bands. (B) Time-dependent O–D (left axis) and O–H (right axis) peak/foot ratios during exposure to D_2_O-humidified N_2_, indicating progressive H/D exchange in Mg­(OH)_2_. (C) Ca­(OH)_2_ spectra collected under identical conditions show reduced O–H intensity after D_2_O exposure but no discernible O–D band, signifying the absence of H/D exchange. (D) Normalized intensities of the broad D_2_O film feature (≈2524 cm^–1^) and hydroxide O–H band (≈3654 cm^–1^) versus exposure time. A baseline shift occurs upon D_2_O introduction, affecting overall intensity but not chemical composition. The increase in the 2524 cm^–1^ feature reflects adsorbed D_2_O film formation.

### Data Modeling

To interpret the SANS results, we employed a hierarchical scattering model^20^ comprising (i) nano- and mesoscale log–normal particle populations, (ii) a surface-fractal component, and (iii) a water-film term modeled through core–shell scattering. Such hierarchical structures (Figure 3) are typical of powder aggregates and have been described by similar multiscale scattering models.^21^

**Figure 3:**
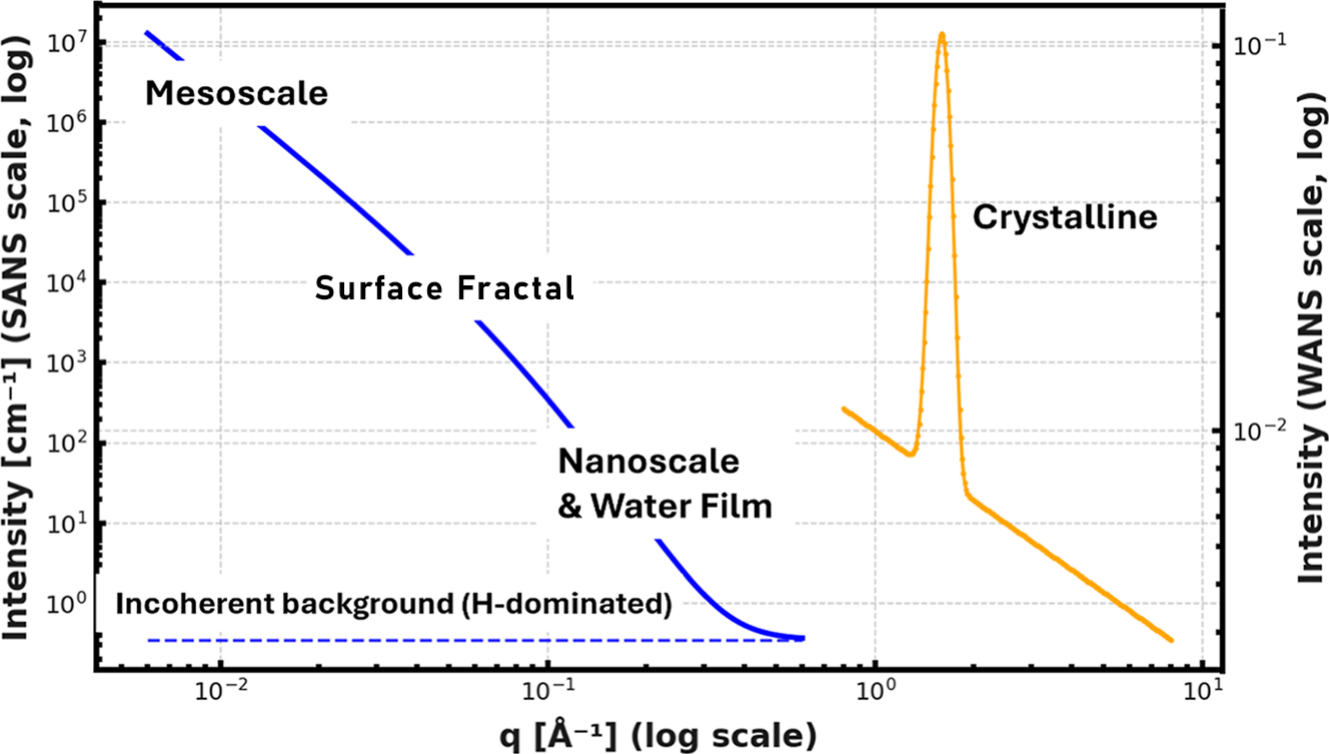
Illustration of q dependence for SANS (blue) and WANS (yellow) scattering. In the SANS region, the hierarchical structure is expressed by the power-law-like behavior in the labeled q ranges, truncated at high q by the background. This background is dominated by incoherent scattering from hydrogen, which appears as a q-independent signal and serves here as a quantitative measure of hydrogen content. In the Supporting Information section, Hierarchical Structure Model for SANS, we discuss the use of the poly disperse size determination in IRENA^22^ to define model parameters. The WANS scattering is dominated by Bragg scattering from crystalline material. As carbonation proceeds, scattering from the dominant peaks for Mg­(OH)_2_ and Ca­(OH)_2_ decreases.

The LogNormal particle model describes scattering from a population of approximately spherical particles whose radii follow a log–normal size distribution. The scattering intensity is obtained by integrating the single-particle form factor over this distribution, using inputs that include the median radius, standard deviation (σ) defining the distribution width, particle contrast (Δρ), and volume fraction.

The surface fractal model characterizes scattering from materials whose interfaces are rough or irregular across multiple length scales. There are four key inputs: the particle contrast (Δρ); S_0_, which scales with the total interfacial area; ξ_s_, which defines the length scale over which surface roughness is correlated (at low *q*, the intensity exhibits a smooth roll-over near *q* ≈ 1/ξ_s_); and the fractal dimension *D*_s_, which quantifies the degree of surface roughness (2 ≤ D_s_ ≤ 3, where *D*_s_ = 2 corresponds to a smooth interface following a Porod *q* ^–4^ power law). We utilize surface roughness, *S*_sf_ = *S*_0_(ξ_s_/*R*_c_)^(D_s_−2)^ to summarize the surface fractal structure.

A core–shell structure is used to model the water film. Utilizing a LogNormal particle model with large radius such that *qR* ≫ 1and having null scattering for interior and exterior, i.e. a vesicle, the water film is characterized by thickness, volume fraction, and SLD. The model is equivalent to a water film coating a solid particle, but relieves the constraint of its volume being dependent on surface area of the particles.

The neutron scattering length densities (SLDs) of the solids and liquids used in this study are listed in Table S4. Because reaction alters the solid composition, neutron contrast will change in one of two ways: For H-D substitution, the resulting single-phase solid is the mole-fraction weighted sum of the composition end members. For two-phase materials, we follow the convention from Allen,^6^ that gives overall contrast as the mole-fraction weighted (his term α) sum of the end-member contrasts. The resulting values of Δρ,^2^ calculated by combining reaction extent (which gives α) are provided in Tables S9–S14.

Given the model’s complexity, parameter constraints were applied to ensure convergence to physically meaningful values. Utilizing the polydisperse size distributions obtained in IRENA^22^ (Figures S4 and S5), refinement of the nano- and mesoscale particle populations was limited within those size ranges.

The optimized parameters are summarized in Tables S9–S14. Uncertainties were estimated using Monte Carlo perturbation (20 cycles) within IRENA, yielding typical standard deviations of 1–6%, with the upper value consistent with data spread and adopted throughout all plotted results. Complementary DLVO-hydration force calculations of equilibrium film thickness (Table S5) provided theoretical context for the experimentally observed water-film growth and isotope dependence.

When scattering in the high q SANS range is in the terminal regime: the Porod theory applies:^23^ $I\left(q\right)=\frac{C_{P}}{q^{4}}+BGD$ , where *C*_P_ is the Porod constant, proportional to surface area and BGD is the *q*-independent background, dominated by incoherent scattering from hydrogen.

Attempts to fit the high-*q* scattering data to the terminal Porod function were found to be problematic in deriving reliable values of for *C*_P_.

Across multiple *q*-ranges (*q*_min_ = 0.2–0.4 Å^–1^; *q*_max_ = 0.45–0.57 Å^–1^), the fitted power-law slopes from plots of log­(I – BGD) versus log­(*q*) did not consistently equal −4, as would be expected in the Porod regime.^18^ This inconsistency reflects the sensitivity of the high-*q* region to both background level and noise from counting statistics, making *C*_P_ values unreliable for these materials.

In contrast, the background (BGD) parameters are well behaved and reproducible. For all *q*-ranges examined, the derived backgrounds varied little between fits and between data sets. Typical uncertainties are ±0.003–to-0.008 cm^–1^ st^–1^, (or 2-to-5%) confirming that the incoherent background determination is well constrained. Values of *q*_min_ = 0.2 Å^–1^ and *q*_max_ = 0.45 Å^–1^ are used throughout for the reported values here.

## Results and Discussion

### Formation and Structure of Water Films

This section establishes that nanometer-scale interfacial water films form readily on both hydroxides under humidified conditions, but differ substantially in thickness, stability, and isotopic response between Ca­(OH)_2_ and Mg­(OH)_2_.

The onset of water adsorption on hydroxide surfaces was examined under humidified N_2_ prior to carbonation. Because hydrogen dominates neutron incoherent scattering, variations in the Porod background provide a direct quantitative measure of changes in hydrogen contentwithin the illuminated volume and thus serve as a sensitive probe of interfacial water. As shown in Figure 4, both Ca­(OH)_2_ and Mg­(OH)_2_ exhibit measurable increases in background intensity upon exposure to H_2_O vapor, indicating the formation of adsorbed films. Calculations from the background variation yield water-film thicknesses of approximately 0.1 nm for Ca­(OH)_2_ and 0.5 nm for Mg­(OH)_2_ (Table S6). These correspond to the initial quasi-liquid layers expected at relative humidities above 70%, consistent with prior adsorption studies of hydroxide surfaces.

**Figure 4:**
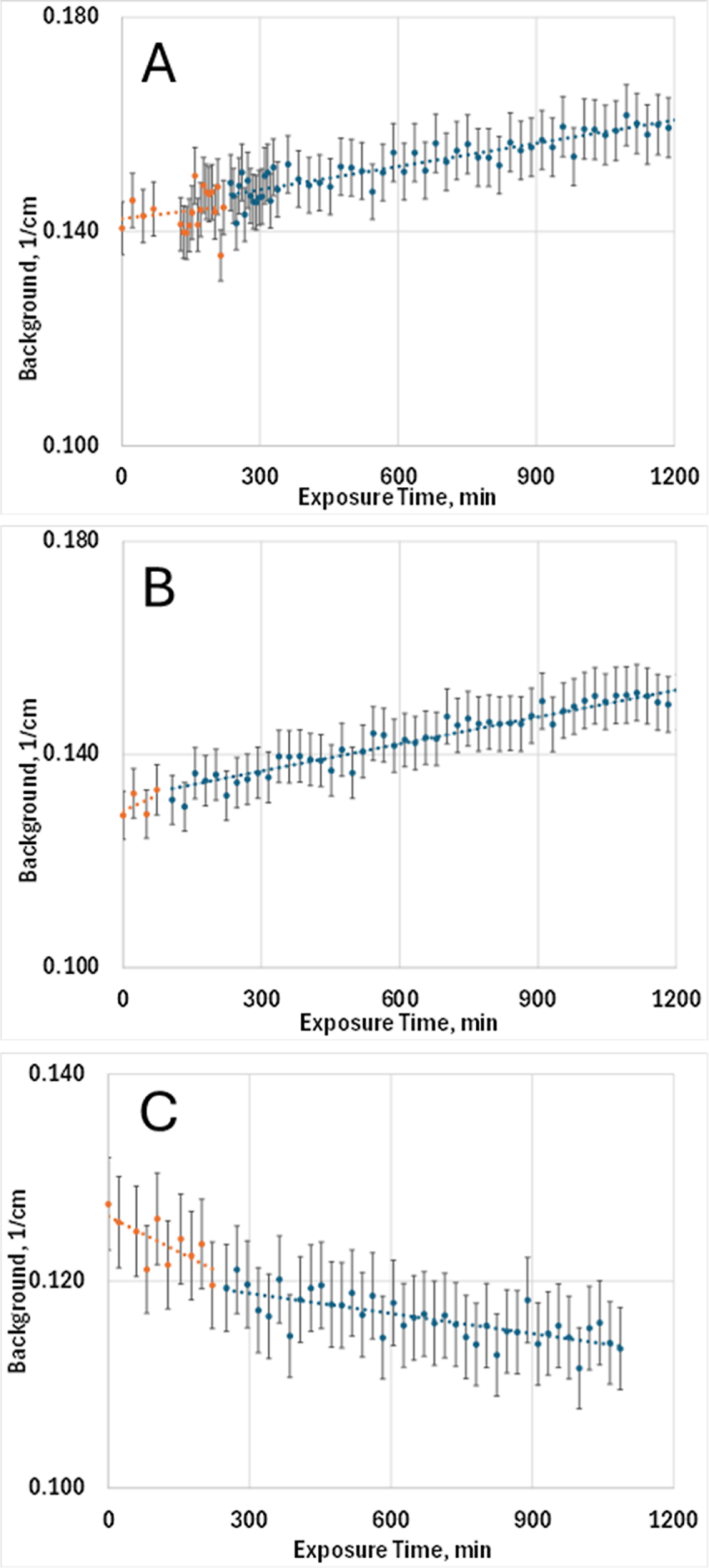
Variation of background intensity during exposure, reflecting contributions from water-film formation (orange) and carbonation product formation (blue). Samples and treatments are (A) CH1_N2_H2O and CH1_H2O_CO2, (B) MH1_N2_H2O and MH1_CO2_H2O, and (C) MH4_N2_D2O and MH4_CO2_D2O. Recalling that background is dominated by hydrogen incoherent scattering, for H_2_O-based systems, the increase in background arises from water-film growth, whereas for D_2_O-based systems, the decrease reflects D–H exchange. Under CO_2_ exposure, increases correspond to hydrated product formation in H_2_O, while decreases indicate formation of hydrated D_2_O carbonates. Dashed lines are linear fits. Error bars represent ±3.5%.

Confirmation of film formation is further supported by intensity-ratio analysis comparing humidified and dry conditions. As shown in Figures S2 and S3, the observed *q*-dependent intensity enhancement matches predictions from a core–shell model, with the effect most clearly resolved in D_2_O due to its higher contrast. These results independently corroborate the film thicknesses derived from background analysis.

To assess whether the measured thicknesses align with theoretical expectations, a continuum force-balance model combining DLVO and short-range hydration interactions was employed (Table S5). Calculated equilibrium film thicknesses at 90% RH are ∼0.5 nm, increasing sharply with humidity. These are similar to the values from background variation but somewhat smaller than the D_2_O film thickness obtained for our best-resolved system MH4_CO2_D2O. (That film’s thickness evolution is discussed below). It is possible the larger value is constrained by D_2_O hydrogen bonding and surface tension.

When Mg­(OH)_2_ is exposed to D_2_O vapor (sample MH4_N2_D2O), the Porod background decreases rather than increases, signifying loss of hydrogen from the scattering volume. This unexpected trend, corroborated by PM-IRRAS, arises from partial H–D exchange at the surface hydroxyls. Despite this isotopic substitution, SANS modeling still detects progressive growth of a D_2_O shell with time (Figure S10), with fitted film thicknesses approximately 1.3 nm at late times (Table S12)

Collectively, the background variation, intensity-ratio analysis, and theoretical modeling establish that nanometer-scale water (and deuterated-water) films form readily on Ca­(OH)_2_ and Mg­(OH)_2_ surfaces under humidified gas flow. These films provide the reactive medium for subsequent isotope exchange and carbonation processes described in the following sections.

These observations demonstrate that interfacial water films are not passive adsorbates but constitute chemically distinct reaction environments whose properties depend strongly on cation hydration.

### Isotopic Exchange as a Probe of Surface Reactivity

This section uses hydrogen–deuterium exchange as a direct chemical probe of surface accessibility and hydration dynamics, revealing fundamental differences in reactivity between Mg­(OH)_2_ and Ca­(OH)_2_.

The background decrease observed for Mg­(OH)_2_ under D_2_O exposure (sample MH4_N2_D2O, Figure 4) provided the first indication of hydrogen–deuterium exchange at the solid–film interface, an unexpected result.^24,25^ Because incoherent neutron scattering from hydrogen dominates the high-q background, even small changes in hydrogen content can be detected quantitatively. Linear interpolation of the background evolution (Table S7) yields a composition corresponding to 0.96 Mg­(OH)_2_ + 0.04 Mg­(OD)_2_ after 220 min of D_2_O exposure, demonstrating that approximately four percent of surface hydroxyl groups were replaced by deuterium.

PM-IRRAS measurements confirm this isotopic substitution spectroscopically. As shown in Figure 2, the O–H stretching band at 3700 cm^–1^ diminishes in intensity while a new O–D stretch appears at 2727 cm^–1^. The temporal evolution of their relative intensities (Figure 2B,D) exhibits a rapid initial exchange phase that slows after ∼8 h, consistent with surface saturation. The absence of any corresponding change in the carbonate or carbonyl regions (1300–1800 cm^–1^) verifies that this process occurs independently of CO_2_ exposure.

Quantitative tracking of the O–H/O–D peak ratios, derived from the PM-IRRAS “peak/foot” intensity method described in Figure 2B, provides a second independent measure of exchange kinetics. The kinetic curves mirror those extracted from the SANS background analysis, confirming that the observed neutron contrast changes are due to real isotopic substitution rather than humidity variation.

No O–D band is observed for Ca­(OH)_2_ under identical D_2_O exposure (Figure 2C), indicating that its hydroxyls are less labile and that the surface is effectively nonexchangeable under these conditions. This contrast between the two hydroxides emphasizes that Mg­(OH)_2_ surfaces possess dynamically hydrated, chemically accessible sites capable of isotopic substitution, while Ca­(OH)_2_ does not. The D_2_O films therefore not only mediate physical adsorption but also enable chemical exchange with the substrate, providing a mechanistic link between hydration environment and surface reactivity.

The presence of H–D exchange on Mg­(OH)_2_ but not on Ca­(OH)_2_ provides direct evidence that differences in interfacial hydration and surface accessibility underlie their divergent carbonation pathways.

### Identification of Carbonation Products

This section identifies the chemical nature and hydration state of the carbonation products, demonstrating that both hydroxides initially form hydrated amorphous intermediates whose subsequent evolution diverges.

Coupled analysis of WANS and SANS data reveals both the phase evolution and chemical identity of the carbonation products. Wide-angle diffraction patterns (Figure S1) show progressive diminution of the (001) reflections of portlandite and brucite with CO_2_ exposure, while no new sharp peaks emerge—indicating that the reaction products are amorphous or nanocrystalline. The reaction extent was quantified from linear fits of the (001) peak-intensity decay (Table S8)

The incoherent background—typically regarded as experimental noise—proved to be a sensitive quantitative reporter of hydrogen content and isotopic exchange during carbonation. The proportionality between background change and composition allows the identity and hydration state of reaction products to be deduced directly from neutron data. This constitutes an in situ chemical fingerprinting method that distinguishes among possible carbonate products based on their hydrogen content, providing evidence that ACC and AMC form under these conditions.

For Ca­(OH)_2_ (CH1_CO2_H2O), the measured background increase is consistent with formation of amorphous calcium carbonate containing approximately 1.6 mol of structural water (ACC·1.58 H_2_O), Figure 6A. In contrast, Mg­(OH)_2_ under H_2_O-humidified CO_2_ (MH1_CO2_H2O) produces a more hydrated amorphous magnesium carbonate (AMC·2 H_2_O), Figure 6B. Under D_2_O-humidified conditions (MH4_CO2_D2O), the same analysis yields a partially deuterated composition, AMC·(1.3 D_2_O + 0.7 H_2_O), Figure 6C, reflecting incorporation of deuterium into the product’s bound water. The complete compositional derivations and neutron-contrast calculations are listed in Tables S9–S14.

**Figure 5:**
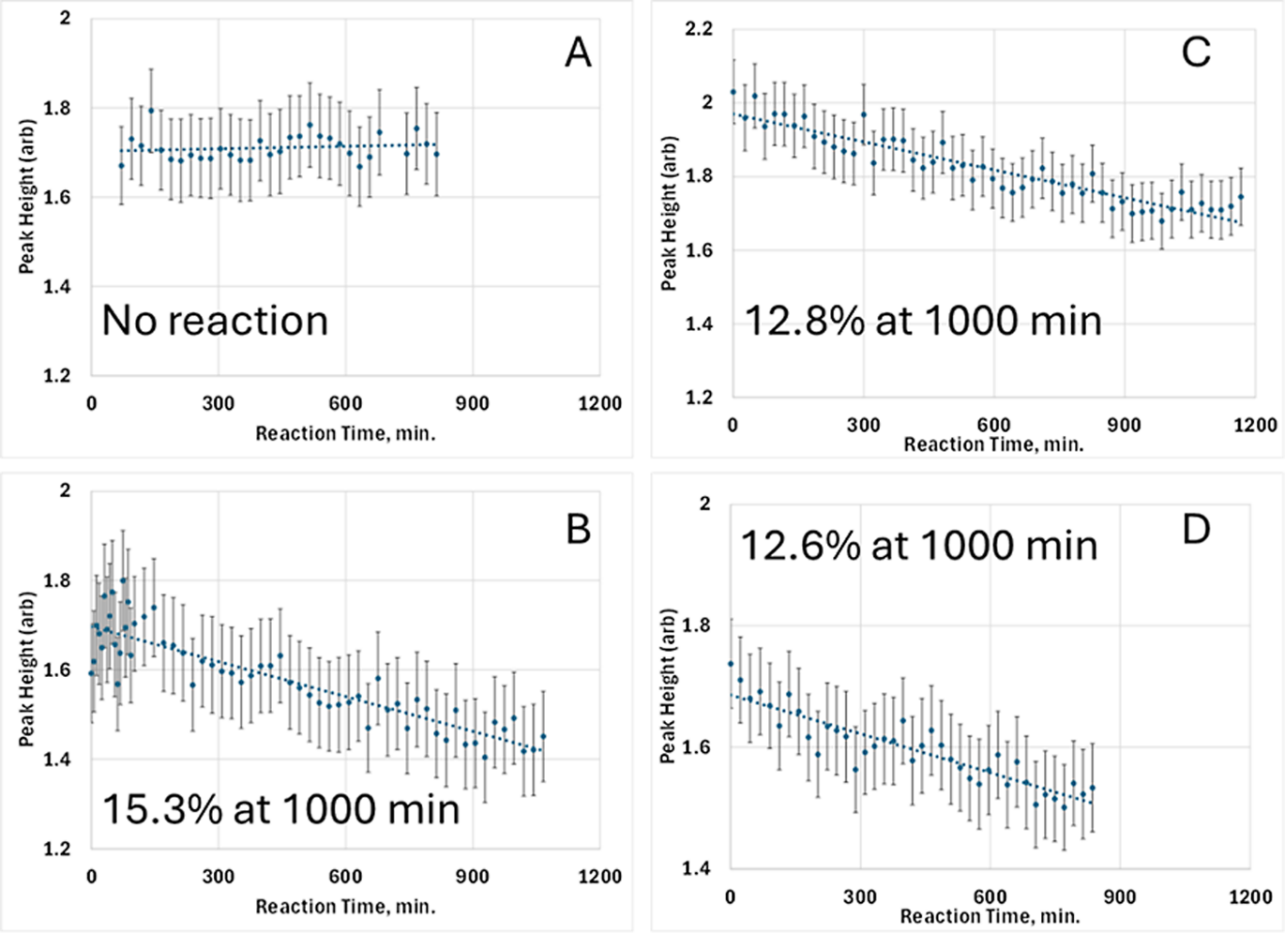
Bragg peak analysis of (001) reflections for Ca­(OH)_2_ and Mg­(OH)_2_ during carbonation. The samples are (A) CH1_CO2, (B) CH1_H2O_CO2, (C) MH1_CO2_H2O, and (D) MH4_CO2_D2O. For Ca­(OH)_2_ and Mg­(OH)_2_, the intensity of the (001) portlandite and brucite peaks, respectively, provides a measure of reactant consumption during carbonation. Linear interpolations (dashed lines) are fit to the data and expressed by equations provided in the Supporting Information, which define the reaction extent used throughout this work. These equations provide the percent reaction at 1000 min shown. As expected from prior studies, exposure to dry CO_2_ produces no measurable reaction. Error bars represent ±1 standard deviation obtained from the peak-fitting routine.

**Figure 6:**
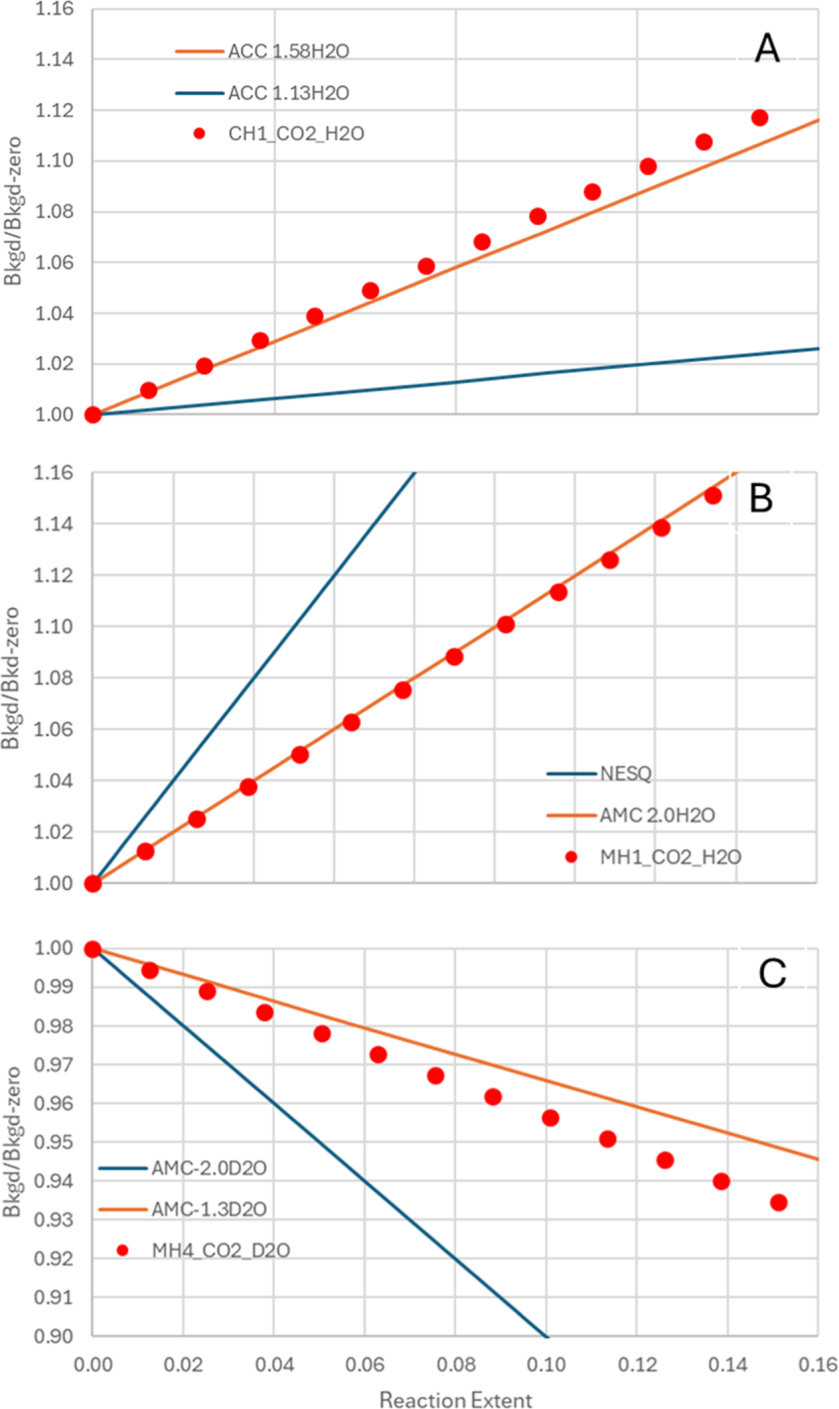
Comparison of measured and predicted background intensity changes during carbonation reactions. (A) CH1_CO2_H2O, (B) MH1_CO2_H2O, and (C) MH4_CO2_D2O. Predicted background variations were calculated using mass-balance relationships (see Supporting Information) and the reaction extent derived from Bragg peak evolution (Figure 5). Experimental background changes (Figure 4) are compared with these predictions to identify reaction products. The calculated compounds correspond to known metastable carbonate phases formed under analogous conditions: ACC, amorphous calcium carbonate; AMC, amorphous magnesium carbonate; and NESQ, nesquehonite.^1,26–33^

These products are consistent with metastable hydrated carbonates reported under low-temperature aqueous carbonation.^1,26–33^ The correlation between background change (Table S7), reaction extent (Table S8), and model compound composition (Table S4) provides quantitative validation that the observed scattering trends arise from transformation of the hydroxides to hydrated amorphous carbonates rather than simple adsorption effects.

The derived hydration levels also reinforce the isotopic dependence established earlier: D_2_O-based experiments yield more stable, thicker interfacial films and correspondingly more hydrated products. The enhanced retention of bound water and slower carbonation kinetics in D_2_O environments support the interpretation that isotope-dependent hydrogen bonding modulates both the structure and dynamics of the carbonation pathway.

Together, the neutron background analysis and reaction-extent measurements establish that interfacial water is incorporated directly into amorphous carbonate products, linking film structure to product identity.

### Nano-to Mesoscale Structural Evolution During Carbonation

This section demonstrates how differences in water-film stability and hydration energetics translate into distinct structural trajectories during carbonation: porosity generation in Ca­(OH)_2_ versus densification and surface smoothing in Mg­(OH)_2_

Quantitative modeling of the SANS profiles using the hierarchical particle model resolves the distinct morphological pathways followed by Ca­(OH)_2_ and Mg­(OH)_2_ during carbonation under both H_2_O- and D_2_O-humidified conditions. Full parameter sets for each experiment are given in Tables S9–S14.

Following the conventions from King,^20^ we use several structural descriptions, including some of which are calculated from the fitted values, in particular: surface areas of the nanoscale and mesoscale particles, mean diameters of those particles, and the rough surface fractal area, *S*_sf_ = *S*_o_(ξ_s_/*R*_c_)^(2–D_s_)^.

For Ca­(OH)_2_ (CH1_CO2_H2O) (Figure 7 and Table S9), carbonation of Ca­(OH)_2_ under humidified CO_2_ produces substantial structural growth driven by formation of an amorphous calcium carbonate (ACC) phase. The mesoscale population increases steadily in diameter and surface area, contributing strongly to the rising SANS intensity (Figure 9A). Nanoscale surface area remains relatively stable, indicating that Ca­(OH)_2_ dissolution and reprecipitation do not destroy fine structure. The surface fractal area decreases modestly, reflecting some smoothing as ACC accumulates, but fractal contributions remain appreciable throughout. Overall, CH1 shows net surface-area gain and aggregate growth, consistent with the development of a hydrated amorphous carbonate coating.

**Figure 7:**
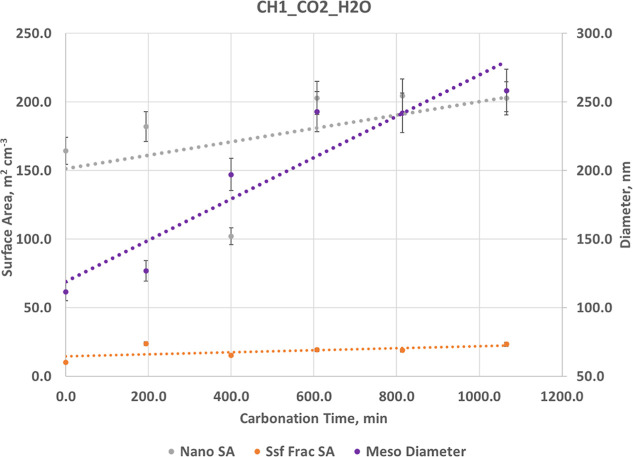
Structural evolution as a function of carbonation time for CH1_CO2_H2O. Increases in nanoscale surface area (i.e., increase in volume fraction) and mesoscale mean diameter. Dashed lines indicate linear trends. Changes are consistent with pore coarsening and structural reorganization. Little change in fractal surface area (*S*_sf_). The maximum reaction extent reached is 15.3%. Error bars are ±5%.

**Figure 8:**
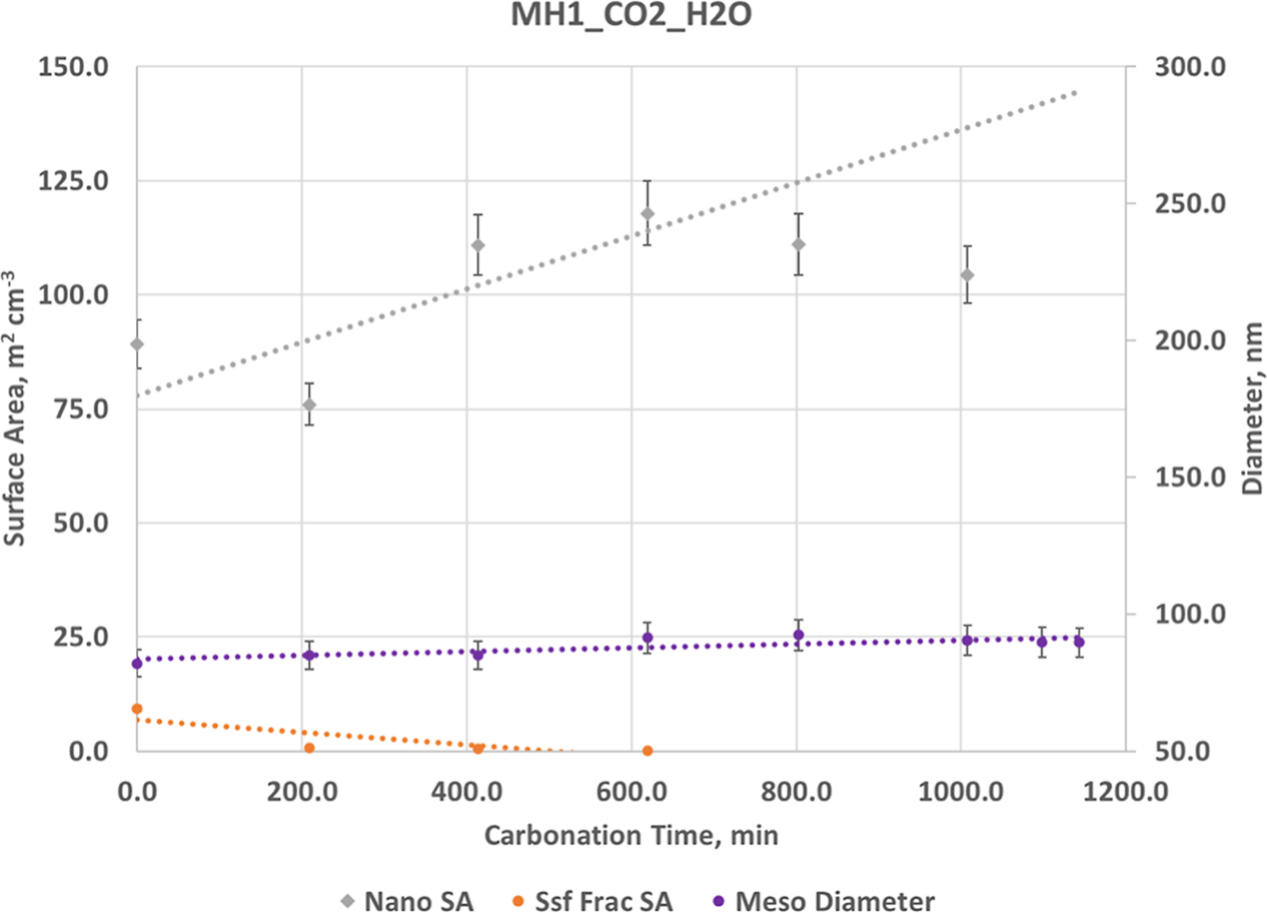
Structural evolution as a function of carbonation time for MH1_CO2_H2O. Increase in nanoscale surface area (i.e., increase in volume fraction) with mesoscale mean diameter showing no significant change. Fractal surface area (*S*_sf_) decreases and goes to zero above 600 min, with no fractal scattering at late times. Dashed lines indicate linear trends. The volume fraction of mesoscale material shows a consistent decrease, that along with disappearance of the fractal component is consistent with densification and loss of mesoscale porosity. The maximum reaction extent reached is 12.8%. Error bars are ±6%.

**Figure 9:**
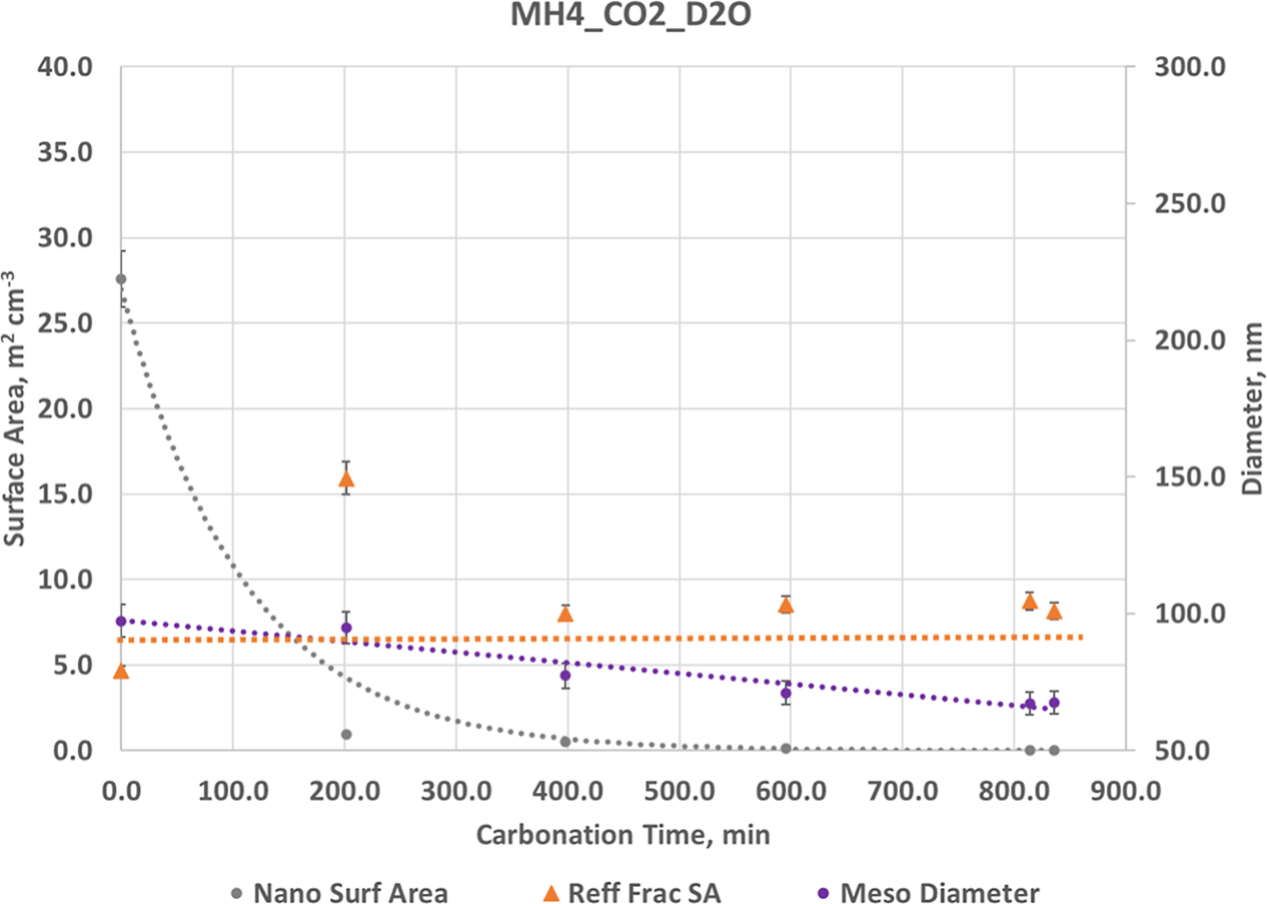
Structural evolution as a function of carbonation time for MH4_CO2_D2O. Decline of nanoscale surface area corresponds to smoothing and densification. A similar indication comes from the mesoscale mean diameter decrease which is accompanied by a decrease in that component’s volume fraction. The rough surface-fractal area, *R*_eff_ (the *S*_sf_ value scaled for contrast change, see text), is small and little changed. Linear and exponential dashed lines are guides to the eye. Like the MH1_CO2_H2O, the structural changes point to a densification and smooth surface structure. The formation of a fluid layer is also resolved, shown in Figure 10 The maximum reaction extent reached is 10.0%. Error bars are ±5%.

The intensity ratio plots in Figure S7A confirm that the largest changes in scattering intensity occur between *q* = 0.01 and 0.1 Å^–1^, the range most sensitive to the development of pore-like features on this length scale.

For Mg­(OH)_2_ (MH1_CO2_H2O) (Figure 8 and Table S10), there is a fundamentally differently behavior from Ca­(OH)_2_. Under humidified CO_2_, the total scattering intensity decreases (Figure S9B) because both mesoscale and fractal surface areas are progressively lost. The mesoscale population remains compact with only minor diameter changes, and nanoscale area diminishes slightly as brucite surfaces smooth. The nanoscale volume fraction increases modestly, consistent with the growth of dense carbonate nuclei that gradually fill or smooth the existing pore space. The absence of measurable change in meso-domain size implies that carbonation proceeds primarily by surface densification rather than particle coarsening. The most significant change is the sharp decline of the surface fractal *S*_sf_ component, which effectively disappears; this loss of interfacial roughness is the dominant driver of the observed intensity decrease. Carbonation leads to surface smoothing and loss of high-surface-area features.

The intensity ratio plots in Figure S7B help illustrate the substantial decline in scattering intensity between *q* = 0.01 and 0.1 Å^–1^, as the surface-fractal scattering transitions to a smooth surface.

For Mg­(OH)_2_ (MH1_CO2_D2O) (Figure 9 and Table S11), there is a fundamentally differently behavior. The total scattering intensity substantially increases across the entire q range (Figure S9C) due to the combined effects of a deuterated solid reaction product along with a D_2_O film. Strong changes in contrast can obscure true structure changes. We use the poly disperse sphere model to help unravel this.

Polydisperse sphere analysis of the MH4_CO2_D2O data set over carbonation times resolves three characteristic scattering populations: a mesoscale population at several tens of nm, an intermediate population centered near 7–8 nm, and a nanoscale population at approximately 3–4 nm. In addition, a broad, continuous distribution between the meso- and nanoscale peaks represents surface-fractal roughness mapped into an equivalent size distribution.

As carbonation proceeds, the intermediate ∼8 nm population becomes increasingly prominent and persists throughout the reaction, consistent with the formation of a D_2_O-rich interfacial film (occurring at a fictive size due to the assumed spherical model see text in Supporting Information and Figure S2). In contrast, the nanoscale population decreases substantially with time, showing an overall reduction of approximately a factor of 2–3 in integrated contribution from the earliest to the latest time point. The mesoscale population evolves more modestly over the same interval. These trends indicate a strong redistribution of scattering intensity from the nanoscale toward the intermediate length scale during carbonation and D_2_O film formation.

Changing contrast also significantly affects the surface fractal model scattering. We note that *S*_sf_ jumps from 5.5 m^2^ cm^–3^ in MH4_N2_D2O to ∼26.5 for MH4_CO2_D2O, this after only a few minutes CO_2_ exposure. It remains at anomalously large values throughout carbonation, strongly suggesting a contrast change effect. Consider the possibility that the surface is covered with AMC MgCO_3_* (1.3D_2_O+0.7H_2_O). Then we can calculate *R*_eff_ = *S*_sf_Δρ_blend^2^_/Δρ_AMC^2^_, where the blend contrast is ratioed to the much larger value for deuterated AMC. The adjustment for our example is 0.175 and *R*_eff_ = 4.7 m^2^ cm^^–3^. This adjustment was applied to all data in Table S11 and Figure 9, and the resulting *R*_eff_ values reflect an unchanging and relatively smooth surface.

The nanoscale volume fraction decreases exponentially at early times, reflecting rapid reaction at accessible surface sites, mimicking the trend found in the poly disperse sphere model. The mesoscale surface area declines linearly throughout the exposure.

The overall structural changes are similar to MH1_CO2_H2O, a smoother, dense layer. These similarities suggest that isotopic substitution modifies contrast and interfacial structure without altering the intrinsic tendency of Mg­(OH)_2_ surfaces toward densification.

Modeling of the D_2_O water film scattering yields an exponential increase in film thickness, following *d*(*t*) = *d*_∞_(1 – *e* ^–*kt*^), with *d*_∞_ = 1.74 nmand *k* = 0.0054 min^–1^ (Tables S11 and S12). Comparison of noncarbonating (MH4_N2_D2O, Figure S10) and carbonating (MH4_CO2_D2O, Figure 10) shows that the film thickens to a higher final thickness (∼1.9 nm) in the presence of CO_2_. Although the time dependence helps explain the evolution, this behavior may reflect coupling between water adsorption and carbonate formation at the interface, where D_2_O films act both as reactants and transport media. Although the poly disperse sphere modeling exhibits the tendencies for film growth and nanoscale population decline, nevertheless we were concerned about possible coupling in the modeling. Therefore, we fixed the nanoscale population and varied the film parameters. The resulting fit reproduced the same time-dependent behavior but gave film thicknesses approximately 30–35% larger, with no improvement in fit quality. This suggests that the original model, with both components refined simultaneously, provides a more physically realistic description of the D_2_O film evolution, as it preserves the total scattering intensity balance and avoids systematic overestimation of the film thickness.

**Figure 10:**
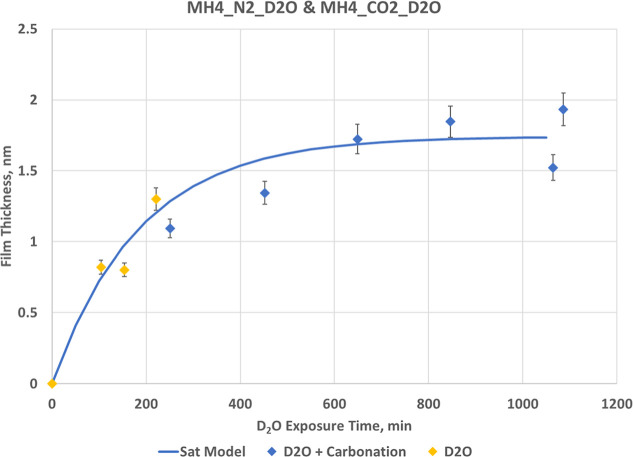
Film thickness evolution derived from the vesicle scattering model for initial D_2_O adsorption on MH4_N2_D2O and, continued growth during carbonation MH4_CO2_D2O. The results exhibit a behavior well described by an exponential model, *d*(*t*) = *d*_∞_(1 – *e* ^–*kt*^), yields *d*_∞_ = 1.74 nm and *k* = 0.0054 min^–1^. Error bars represent ±6%.

The following analysis provides an order-of-magnitude physical context for D_2_O film evolution. Using the fit parameters from the exponential model, interpreting k as a transport rate constant (τ = 1/*k* ≈ 3.1 h), and using τ ≈ *L* ^2^/π^2^ *D*_eff_, implies an effective diffusivity *D*_eff_ ≈10^–12^–10^–13^ m^2^/s for characteristic length scales *L* ≈ 30–100 μm. This is consistent with a tortuous, partially condensed pore network, supporting a diffusion-limited equilibration of the D_2_O film rather than a molecular-kinetic limitation at the interface.

Overall, isotopic substitution does not suppress the intrinsic tendency of Mg­(OH)_2_ surfaces to smooth during carbonation. Instead, D_2_O fundamentally alters the morphology and contrast of the product interface, redistributing roughness from the original brucite surface to the newly formed AMC/D_2_O layer. The contrast-normalized metric *R*_eff_ is therefore essential for separating true structural evolution from contrast-driven intensity amplification in CO_2_–D_2_O environments.

### Mechanistic Framework and Broader Implications

The combined neutron and infrared results converge on a unified mechanistic framework in which interfacial water-film stability governs isotope exchange, amorphous carbonate persistence, and nanoscale structural evolution.

In this framework, neutron scattering and infrared spectroscopy play complementary roles. The neutron data quantitatively define the evolution of interfacial water films, reaction extent, and nano-to mesoscale structural changes during carbonation. The infrared spectra provide independent chemical confirmation, revealing the presence and removal of interfacial water films and uniquely identifying hydrogen–deuterium exchange at the hydroxide surfaces. Together, these measurements define a consistent sequence linking film formation, isotopic substitution, and carbonation-induced structural transformation.

Film Formation: Under humidified N_2_, thin adsorbed water layers (0.1–0.5 nm for H_2_O; 1.3–1.5 nm for D_2_O) develop on hydroxide surfaces. These quasi-liquid films, confirmed by SANS modeling, intensity ratios (Figures S2 and S3), and background analysis (Table S6), provide a continuous medium for proton transport and CO_2_ dissolution.

Isotopic Exchange: On Mg­(OH)_2_, the interfacial D_2_O film participates in rapid H–D exchange, producing up to 4% Mg­(OD)_2_ (Table S12). The absence of exchange on Ca­(OH)_2_ highlights fundamental differences in surface accessibility and hydration bonding between the two hydroxides.

Carbonation Reaction: Introduction of CO_2_ transforms these hydrated surfaces into amorphous carbonate hydrates, as demonstrated by WANS and SANS mass-balance analysis (Figure 6, and Supporting Information Section: Mass Balance Equations for Reaction Products). The resulting products—ACC·1.58H_2_O for Ca, AMC·2H_2_O or AMC·(1.3D_2_O + 0.7H_2_O) for Mg—reflect incorporation of film water into the solid phase. Thus, carbonation proceeds through amorphous ACC and AMC intermediates that diverge in subsequent dehydration or stabilization. The observation of transient amorphous intermediates is consistent with recent work on MgO hydration, where Bonilla-Correa.^34^ reported an amorphous hydroxide precursor preceding brucite formation and associated with reaction-induced fracturing. Although their system involves pure hydration rather than carbonation, the occurrence of such intermediates supports our interpretation of amorphous carbonate formation as a key kinetic step.

Structural Transformation: As carbonation proceeds, Ca­(OH)_2_ evolves toward increasing porosity and surface complexity (Figure 7), whereas Mg­(OH)_2_ transitions to smoother, denser surfaces (Figures 8 and 9, Tables S9–S14). The D_2_O systems exhibit self-limiting film growth and enhanced structural stability, consistent with slower diffusion and stronger hydrogen bonding.

A broader perspective emerges when these in situ findings are considered together with our previous ex-situ study.^20^ There, Ca­(OH)_2_ was found to carbonate directly to CaCO_3_, whereas Mg­(OH)_2_ formed a hydrated carbonate, tentatively identified as nesquehonite. The present in situ measurements reveal that both systems initially form amorphous, hydrated carbonates—ACC for Ca and AMC for Mg—before diverging upon dehydration. The Ca phase spontaneously dehydrates to CaCO_3_ at room temperature, while the Mg phase remains hydrated. This divergence likely reflects the stronger hydration affinity of Mg^2+^ and may also be connected to its enhanced H–D exchange activity observed under these humid conditions. The combined observations establish that the stability of these amorphous intermediates—and thus the ultimate product identity—is controlled by the cation–water affinity and the persistence of nanometric water films.

Beyond these model hydroxides, the implications extend to natural weathering, cementitious materials, and mineral carbonation processes central to the durability of built infrastructure. The observed isotope effects reveal how subtle differences in hydrogen-bond strength and molecular dynamics can influence hydration stability, reaction pathways, and the persistence of amorphous phases. Together, these insights underscore the governing role of interfacial water in controlling mineral reactivity and highlight the capacity of in situ neutron scattering and infrared spectroscopy to quantitatively resolve such processes at buried solid–gas interfaces.

Taken together, the results establish that carbonation pathways in calcium and magnesium hydroxides are governed by the thermodynamic stability and chemical accessibility of nanometer-scale interfacial water films. In calcium systems, thinner, more labile films promote rapid dehydration of initially formed amorphous carbonate, leading to pore generation and increased surface complexity. In contrast, the stronger hydration affinity of Mg^2+^ stabilizes thicker, more cohesive water films that persist under reaction conditions, enable hydrogen–deuterium exchange, and favor retention of hydrated amorphous carbonate phases. These differences at the nanometer scale propagate upward, producing the macroscopic divergence between porosity generation in Ca­(OH)_2_ and densification and surface smoothing in Mg­(OH)_2_. More broadly, the findings demonstrate that interfacial water is not merely a prerequisite for carbonation, but the primary control parameter that links hydration energetics, reaction pathways, and structural evolution during low-temperature mineral transformation.

## Supplementary Material



## Footnotes

1 Certain commercial instruments, materials, software or processes are identified in this paper to specify the experimental procedure adequately. Such identification does not imply recommendation or endorsement by the National Institute of Standards and Technology, nor does it imply that the instruments, materials or processes identified are necessarily the best available for the purpose.

## Notes

Experimental details of samples and gas exposure conditions (Table S1); WANS diffraction results and phase identification (Figure S1); core–shell and vesicle scattering model calculations for thin water films (Figures S2–S3); hierarchical SANS structural modeling and particle size distributions (Figures S4–S7, Tables S2–S3); neutron scattering length densities for model compounds (Table S4); DLVO + hydration disjoining pressure model and isotope-dependent film thickness calculations (Figure S8, Table S5); background-variation analysis and derived H_2_O film thickness (Table S6); mass balance equations for carbonation products and isotope exchange; interpolation equations for background and Bragg peak evolution (Tables S7–S8); full SANS fitting parameters for all reaction conditions (Tables S9–S14, Figures S9–S10); PM-IRRAS spectra under H_2_O and D_2_O humidification (Figure S11) (PDF)
