# Supplementary material for: Studies of Water Films and Carbonation via Neutron Scattering and Infrared Adsorption: In Situ Studies of Mg(OH)2 and Ca(OH)2
Source: J Phys Chem C Nanomater Interfaces. 2026 Mar 24;130(15):5688–700. doi: 10.1021/acs.jpcc.6c00841 (PMC13093474; doi:10.1021/acs.jpcc.6c00841)
Supplement: Supplementary file 1 [file jp6c00841_si_001.pdf]

Supporting Information

Studies Of Water Films and Carbonation Via Neutron Scattering and Infrared Adsorption: In Situ Studies of $Mg(OH)_2$ and $Ca(OH)_2$

Hubert King[1,4*], Ryan Murphy[1], Avery Baumann[2], Robert Dalgliesh[3], Dirk Honecker[3] and Greg Smith[3]

[1] NIST Center for Neutron Research, National Institute of Standards and Technology, 100 Bureau Dr, Gaithersburg, MD 20878, USA.

[2] Material Measurement Laboratory, National Institute of Standards and Technology, 100 Bureau Dr., Gaithersburg, MD 20878 ,USA.

[3] ISIS Pulsed Neutron and Muon Source, STFC Rutherford Appleton Laboratory, Harwell Campus, Didcot | OX11 0QX | United Kingdom

[4] Department of Chemical & Biomolecular Engineering, Center for Neutron Science, University of Delaware, Newark, DE 19716, USA

Contents

Samples and Gas Exposure Conditions 3

WANS Results 4

Water-Film Scattering Model and Data 5

Hierarchical Structure Model for SANS 9

Model Compound Neutron Scattering Length Densities 16

Disjoining Pressure: Water Film Thickness Calculation 18

Water-Film from Background Variation 25

Mass Balance Equations for Reaction Products 26

Interpolation Equations for Background Variation and Bragg Peak Intensity 28

SANS Results 29

CH1_CO2_H2O 30

MH1_CO2_H2O..33

MH4_CO2_D2O..37

MH4_N2_D2O: D_2O film 42

CH1_CO2_D2O: D_2O film...46

MH1_CO2_H2O: H_2O film 49

PM-IRRAS: Carbonation 53

References 55

Samples and Gas Exposure Conditions

Table S1 Summary of samples and gases

Sample	Type	RH %	Sequence #	Duration, min
CH1_CO2	Carbonation	4.8-15.8	74475 - 74542	837.4
CH1_N2_H2O	Film Formation	89.1	74553 - 74584	220.9
CH1_CO2_H2O	Carbonation	83.5-84.5	74585 - 74702	1077.1
CH1_CO2_D2O	Film Formation	84.1*	74711 - 74720	111.1
MH1_N2_H2O	Film Formation	95.1	74725 - 74732	73.4
MH1_CO2_H2O	Carbonation	89.4-87.3	74733 - 74837	1189.2
MH4_N2_D2O	Film Formation	97.6-98.5*	74842 - 74857	220.8
MH4_CO2_D2O	Carbonation	86.9*	74858 – 74935	852.5

*Note: The RH sensor used was Thorlabs TSP01, which claims accuracy +/- 2% in the range 20-80% RH, and +/- 4% accuracy in the 0-20% and 80 % to100 % range, all for H_2O. To calibrate for D_2O, we compared H_2O and D_2O saturated air, finding that $RH_{D2Otrue}=RH_{D2Oobs}*1.055$, an adjustment applied to table values.

WANS Results

Diffraction peaks from WANS measurements provide structural clues to reaction products and phase identification. Our previous work[1] had identified minor contaminates in the CH sample consisting of calcite and a calcium oxide with diffraction peak at q≈0.6 $Å^{-1}$ These are also seen here. However, the majority phases are portlandite and brucite.

No diffraction peaks from carbonate crystals implies ACC and AMC products rather than the formation of crystalline carbonate phases.

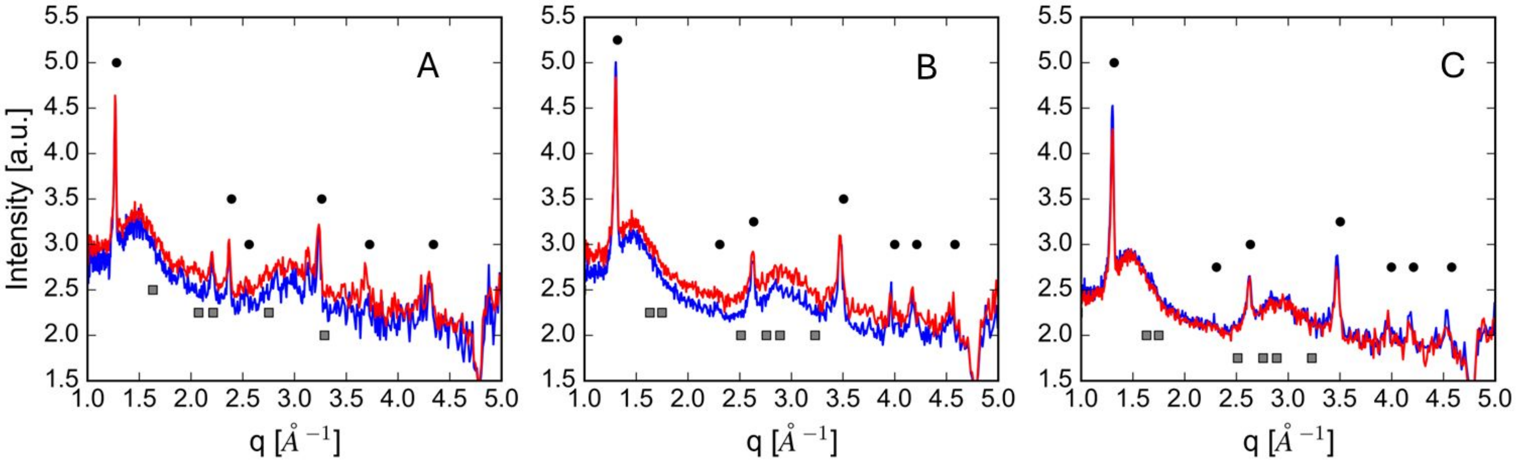

Figure S1 Diffraction data before (blue) and after (red) carbonation under humidified CO_2.(**A**) CH1_CO2_H2O: Diffraction peaks marked above and below correspond to the majority phase, portlandite (•), and a minor phase,

calcite (▪), respectively. During exposure to CO_2 and H_2O, portlandite persists with decreasing peak intensities, while calcite remains unchanged. The absence of new diffraction peaks indicates formation of an amorphous carbonate phase, likely amorphous calcium carbonate (ACC), inferred from the evolving background intensity.(**B**) MH1_CO2_H2O: Major reflections (•) correspond to brucite, with not-observed nesquehonite peak positions by (▪) . No new crystalline phases appear during carbonation, consistent with the formation of amorphous magnesium carbonate species (AMC). (**C**)MH4_CO2_D2O:* Similar brucite (•) and not-observed nesquehonite (▪) peak positions marked. Peak intensities evolve modestly during carbonation, again without new crystalline reflections, supporting formation of AMC. For each, we analyze the peak areas of the most-intense peak (001) to obtain extent of reaction. (see **Figures 5 and Tables S9-S14**).

Water-Film Scattering Model and Data

Here we use a Core-Shell model from SASVIEW [2]. We start with polydisperse sphere sizes distributed with a LogNormal distribution and mean diameter/standard deviation similar to those for both MH and CH samples. We then apply a water film of 5 Å and 10 Å. Owing to the significant differences in neutron scattering length density for H_2O (-0.561×10^{14} m^{-2}) and D_2O (6.393×10^{14} m^{-2}), the ratio of scattering with core-shell to that with no shell, show a considerable difference between H_2O and D_2O and the film scattering is concentrated at high q (**Figure S3**).

S6

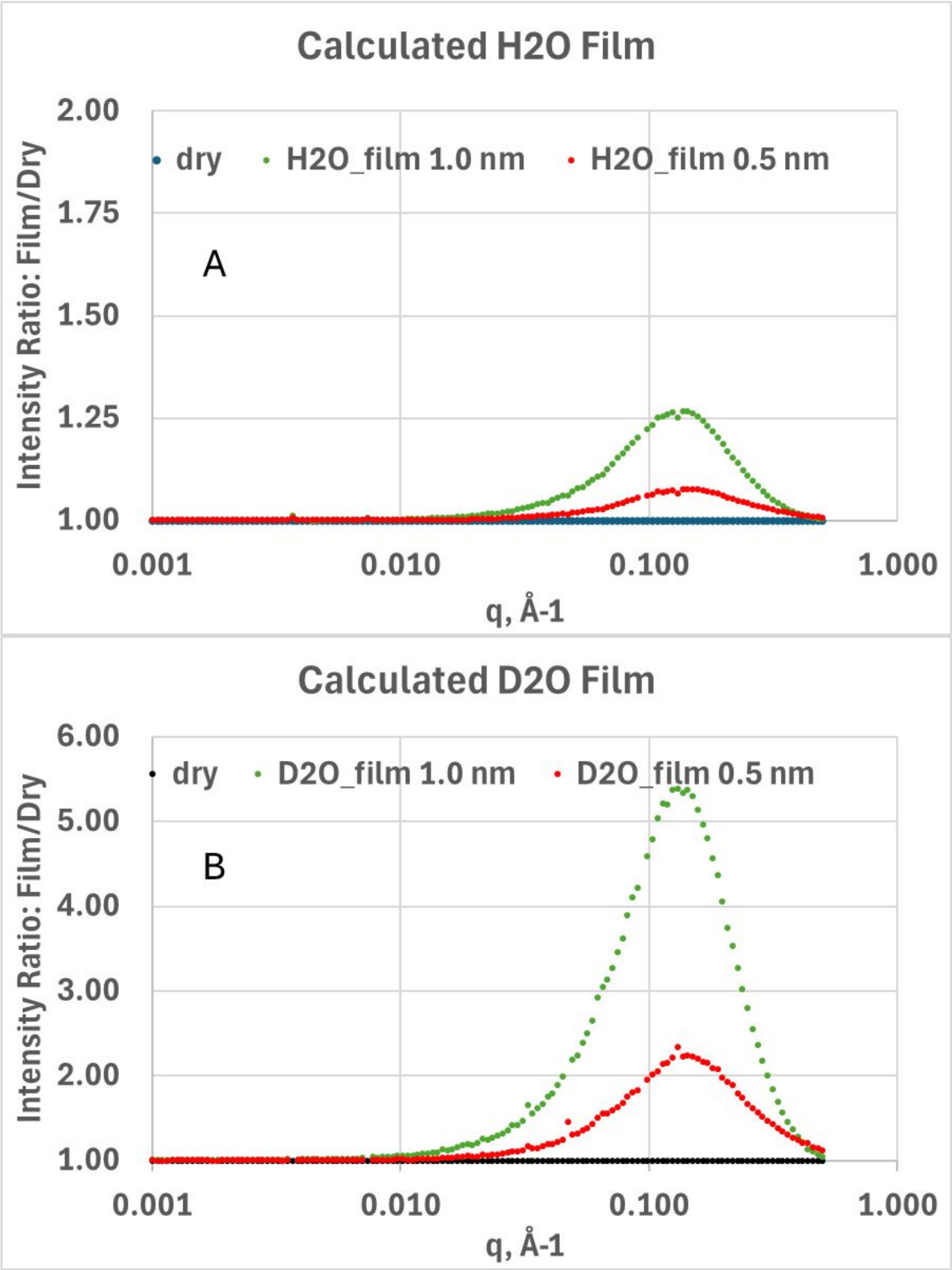
Calculated H2O Film
dry
H2O_film 1.0 nm
H2O_film 0.5 nm
A
Intensity Ratio: Film/Dry
2.00
1.75
1.50
1.25
1.00
0.001
0.010
0.100
1.000
q, Å-1
Calculated D2O Film
dry
D2O_film 1.0 nm
D2O_film 0.5 nm
B
Intensity Ratio: Film/Dry
6.00
5.00
4.00
3.00
2.00
1.00
0.001
0.010
0.100
1.000
q, Å-1

Figure S2. A thin adsorbed water film on a solid particle is modeled using a core–shell form factor in SASView. The plotted ratio of the calculated intensity for film-coated particles to that of the dry particle highlights the scattering contribution of the water film. Separate panels show results for (A) H_2O and (B) D_2O, with film thicknesses of 0.5 nm and 1.0 nm compared to the dry case.

The calculated Film/Dry intensity ratios for thin H_2O and D_2O shells exhibit a broad maximum near $q \approx 0.12$ Å^{-1} whose position is essentially independent of film thickness (5–10 Å). This peak should not be interpreted as a characteristic size of the film itself. In the thin-shell limit, the fluid layer acts as a small perturbation on the scattering from the underlying particle, so the Film/Dry ratio highlights the q-range where the dry particle form factor is most sensitive to changes at its surface. Consequently, the position of the maximum is determined primarily by the core radius and sphere form factor (a “sensitivity window”), while the shell thickness and contrast (H_2O vs D_2O) mainly control the *amplitude* of the effect rather than the q-position of the peak. As we see in main text, if we invert this data under an assumption of a spherical scatterer, we obtain a radius $R = 4.49/0.12$ Å$^{-1} \approx 40$ Å. The factor of 4.49 comes from the spherical form factor.

For fitting purposes, we utilize a different, but equivalent model. Relaxing the constraint that films must be commensurate with the particles, we use a vesicle scattering model. With SLD=0 for core and exterior, only the film scattering is present. The characteristic length scale of such a model is the film thickness, with the vesicle diameter assigned a large value to eliminate the unwanted scattering from the vesicle diameter. For a vesicle radius, R= 150 nm, this gives give $q^*R \gg 1$ for low q. Thus, only scattering from the water film contributes in the q window of interest. See discussion of hierarchical scattering model in main text for details on structural components.

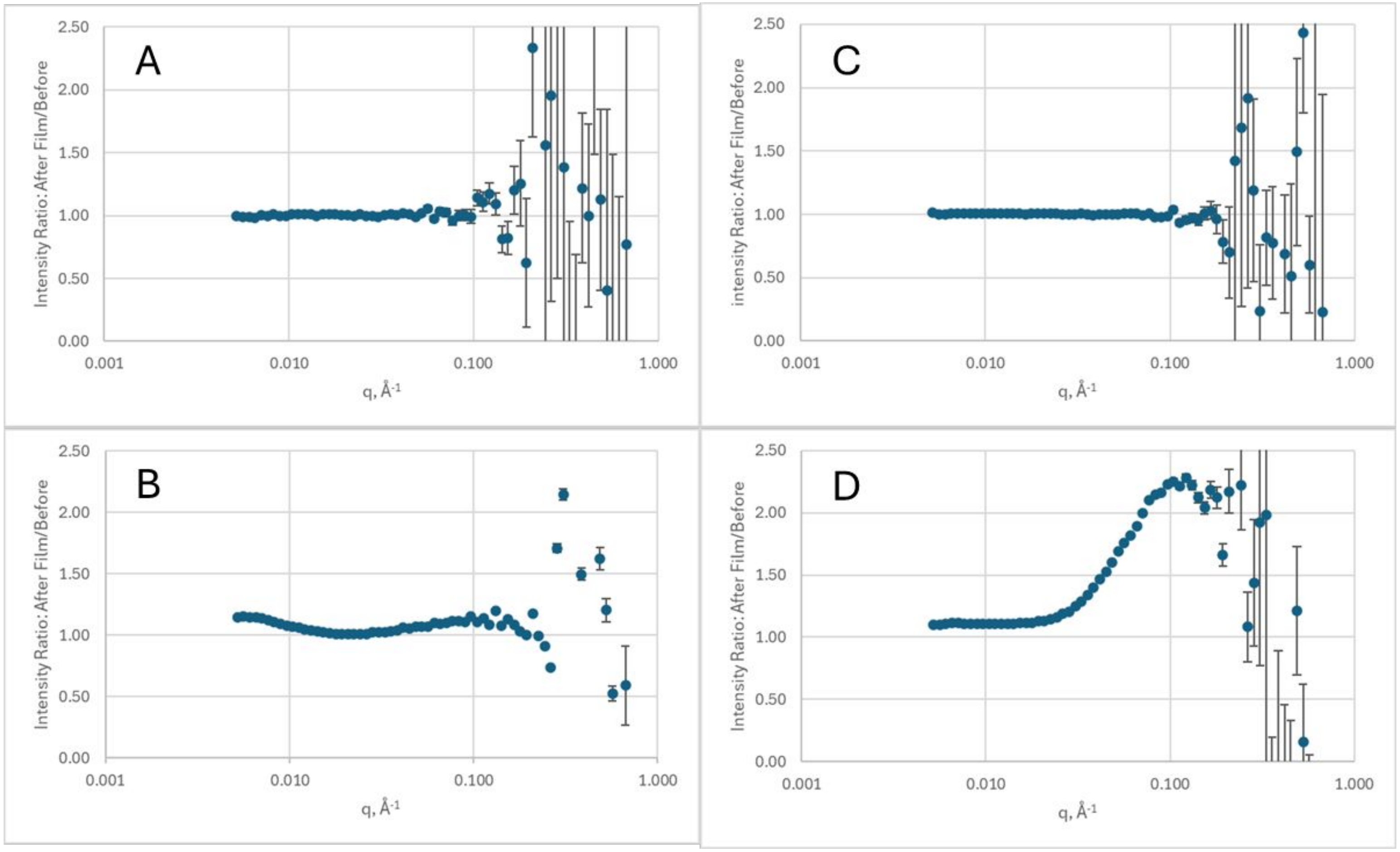

Figure S3 Intensity ratios before and after film formation versus q emphasize excess scattering from water. Samples: (**A**): CH1_N2_H2O, (**B**): CH1_CO2_D2O, (**C**): MH1_N2_H2O, and (**D**): MH4_N2_D2O. Exposure times were, respectively: 221, 111, 73, 221 min. Enhanced scattering in high q range (marginally significant for H_2O)

shows small sizes of films. As expected, the large SLD for D_2O results in significantly more scattering. Compare with calculations from the above core-shell model in **Figure S2**, reasonable agreement. Error bars are ± one standard deviation from experimental intensity.

Hierarchical Structure Model for SANS

We utilize the poly-disperse size determination in IRENA [3] to define initial model parameters for particles. Several examples prior to and after carbonation are given here.

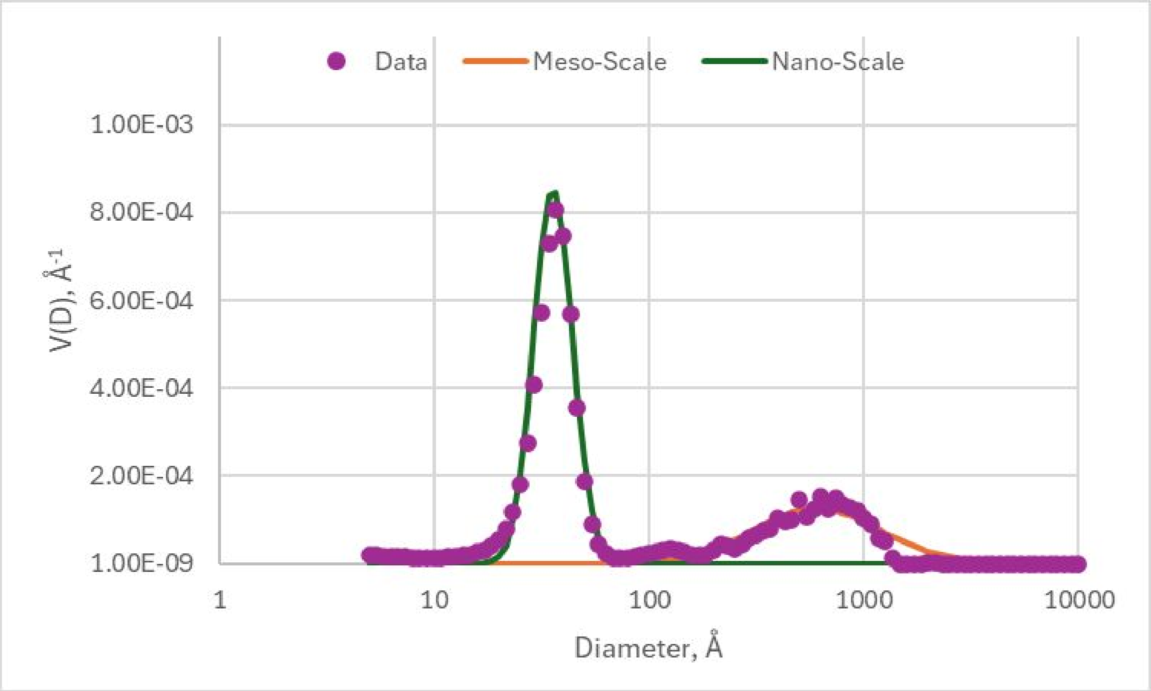

Figure S4. Using the IRENA package (Ilavsky, 2009 #69), a polydisperse sphere model was applied to the CH1_CO2_H2O sample, revealing two characteristic particle size populations—one nanoscale and one mesoscale—with intermediate dimensions represented by a surface-fractal component. Characteristic sizes for each mode are provided in **Table S2**, which serves as the basis for defining the initial model parameters in subsequent analyses.

Table S2 Characteristic sizes for meso-scale and nano-scale models

CH1_CO2_H2O	Meso-Scale	Nano-Scale

Distribution	LogNormal	LogNormal
Mean-Diameter, nm	95.0	3.5
Stdev, nm	0.05	0.005
Scale	0.3	0.075

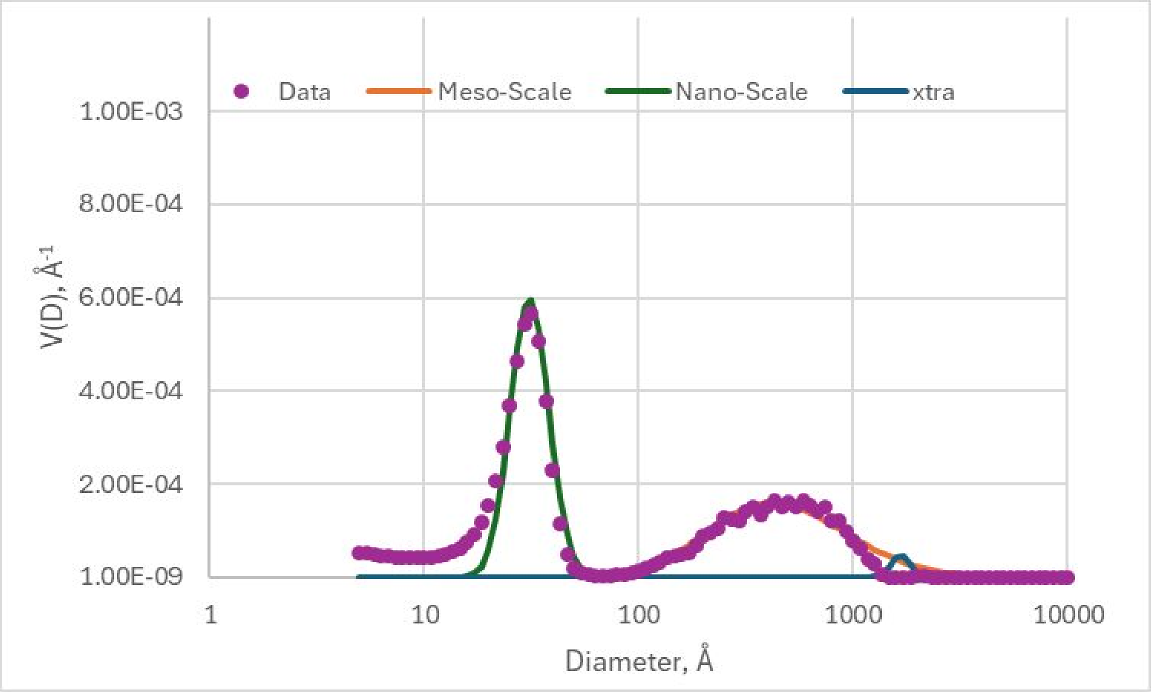

Figure S5. Using the IRENA package (Ilavsky, 2009 #69), a polydisperse sphere model was applied to the MH1_CO2_H2O sample, revealing three characteristic particle size populations—two primary modes in the nano-

and meso-scale ranges, along with an additional large-size component (“Xtra”). Intermediate dimensions are represented by a surface-fractal component. Characteristic sizes for each mode are provided in **Table S3**.

Table S3 Characteristic sizes for meso-scale, nano-scale and Xtra model

MH1_CO2_H2O	Meso-Scale,	Nano-Scale	Xtra*
Distribution	LogNormal	LogNormal	Gaussian
Mean Diameter, nm	75.0	3.0	166.7
Stdev, nm	0.068	0.005	17.5
Scale	0.3	0.045	0.02

*This additional particle size, found only for $Mg(OH)_2$ was previously reported.[1] It is a minor component that gives a low-q scattering signature. Trials where it is not included indicate that the meso-scale scattering model can be distorted. It is unaffected by carbonation.

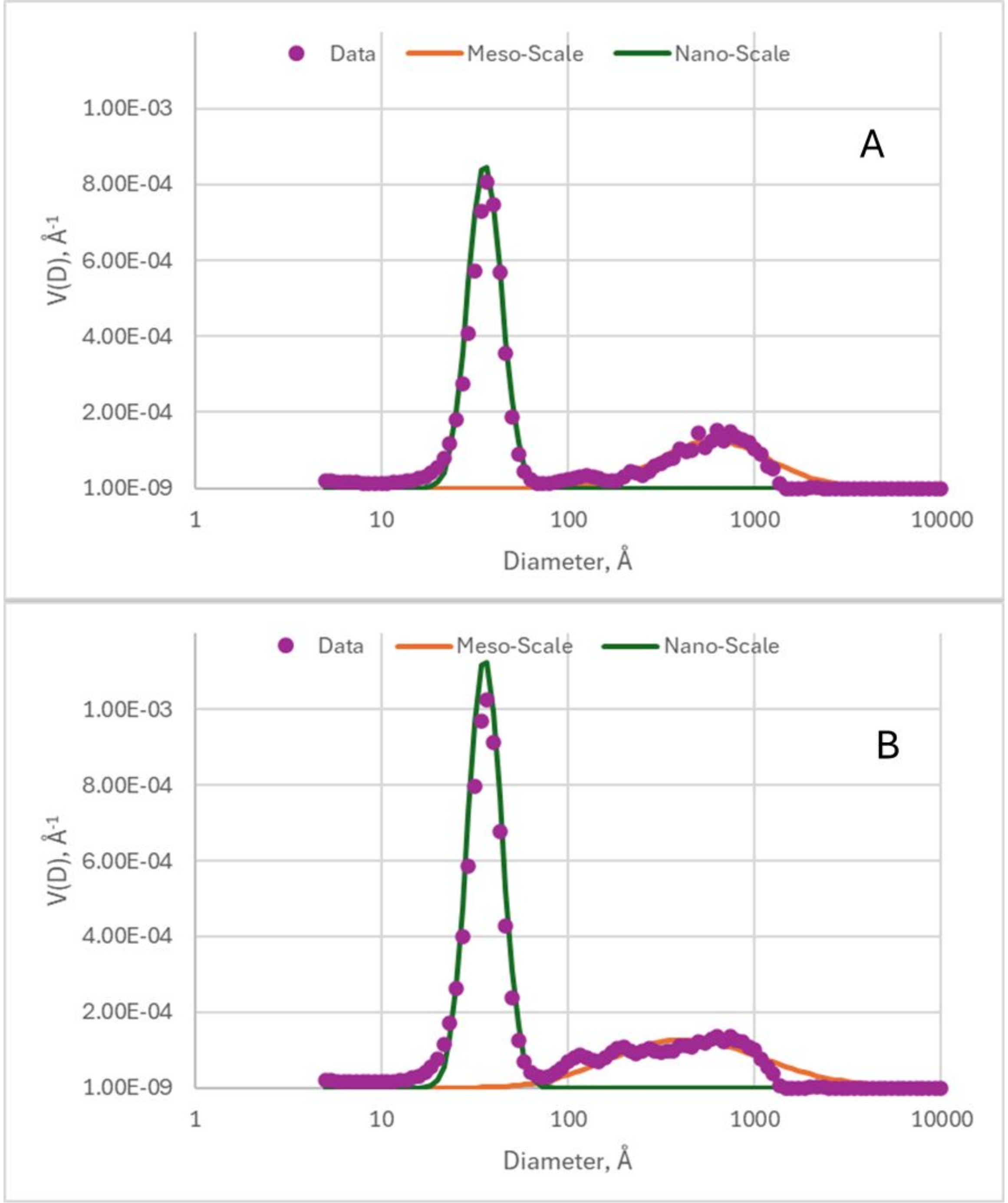
Data
Meso-Scale
Nano-Scale
A
1.00E-03
8.00E-04
6.00E-04
4.00E-04
2.00E-04
1.00E-09
V(D), Å-1
1
10
100
1000
10000
Diameter, Å
Data
Meso-Scale
Nano-Scale
B
1.00E-03
8.00E-04
6.00E-04
4.00E-04
2.00E-04
1.00E-09
V(D), Å-1
1
10
100
1000
10000
Diameter, Å

Figure S6. CH_CO2_H2O sample before carbonation (A) and after 1065.8 min of carbonation (B). Structural parameters used for modeling carbonation were derived from these particle size distribution plots. The results show a pronounced increase in the nano-scale population and greater polydispersity within the meso-scale component following carbonation.

Further insight into the relevant length scales where carbonation impacts structure is gained from the ratio of scattering intensities before carbonation to that afterwards as shown in figures below.

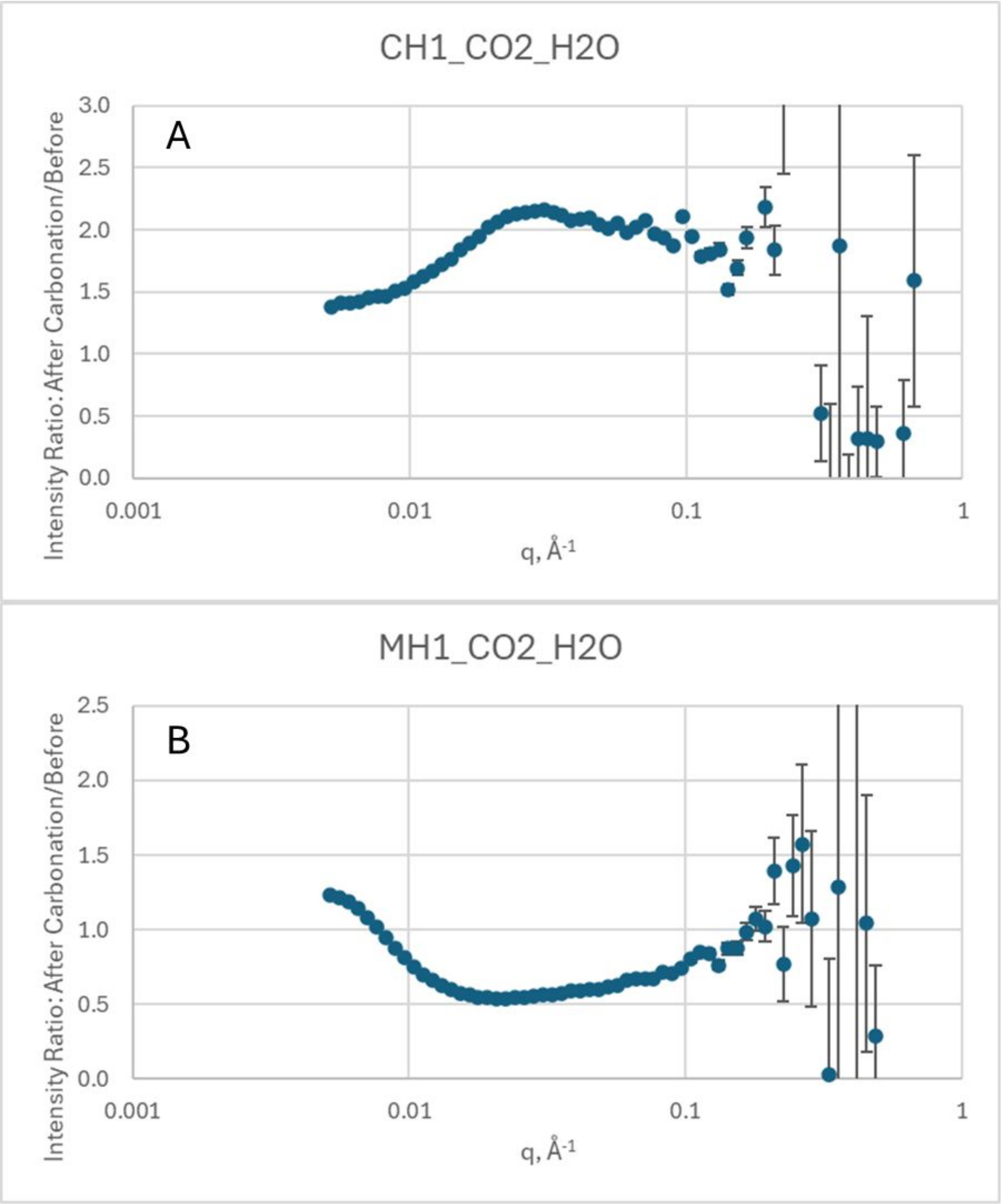
CH1_CO2_H2O
A
Intensity Ratio: After Carbonation/Before
3.0
2.5
2.0
1.5
1.0
0.5
0.0
0.001
0.01
0.1
1
q, Å-1
MH1_CO2_H2O
B
Intensity Ratio: After Carbonation/Before
2.5
2.0
1.5
1.0
0.5
0.0
0.001
0.01
0.1
1
q, Å-1

Figure S7. Ratio of scattering intensities before and after carbonation for (A) CH1_CO2_H2O (t = 0 vs. 1065.8 min) and (B) MH1_CO2_H2O (t = 0 vs. 1161.6 min). Such plots identify the q range over which carbonation alters the scattering, guiding subsequent model refinements. For MH1, the observed decrease in scattering intensity reflects the loss of surface fractal structure during carbonation. Error bars are ± one standard deviation from experimental intensity.

Model Compound Neutron Scattering Length Densities

Table S4 Scattering length densities for model compounds

	density, (g/cm^3)	Mw (g/mole)	Volume (cm^3/mol)	Neutron SLD (10^{14} m^{-2})	Neutron Incoherent Cross-section (cm^{-1} sr^{-1})
Portlandite $Ca(OH)_2$	2.211	74.093	33.511	1.586	3.142
Calcite $CaCO_3$	2.71	100.0869	36.932	4.69	0.005

ACC $CaCO_3$ $1.58H_2O$	2.18	118	54.1	2.666	2.841
ACC $CaCO_3$ $1.58D_2O$	2.18	121.1	54.1	5.880	0.070
Brucite $Mg(OH)_2$	2.34	58.3197	24.923	2.296	4.203
Magnesite $MgCO_3$	2.96	84.3139	28.484	6.224	0.004
Nesquehonite ($MgCO_3 \cdot 3H_2O$)	1.837	138.3	75.286	1.952	4.169
H_2O	1	18.015	18.015	-0.561	5.621
D_2O	1.11	20.028	18.043	6.393	0.138
AMC MgCO3* 2H2O	2.22	120.34	54.1	2.898	3.896
AMC MgCO3* (1.3D2O+0.7H2O)	2.29	123.673	54.1	5.963	1.49
Nesquehonite MgCO3*3D2O	1.84	144.6	78.7153	6.655	0.098
Nesquehonite MgCO3*2D2O*1H2O	1.84	142.6	77.627	5.131	1.49
$Mg(OD)_2$	2.46	60.332	24.525	7.445	0.567

Disjoining Pressure: Water Film Thickness Calculation

To determine the equilibrium thickness of adsorbed water films on hydroxide surfaces such as $Mg(OH)_2$ and $Ca(OH)_2$, we employ a continuum force balance model that combines classical DLVO theory (van der Waals and electrostatic double-layer interactions) with a short-range hydration force. This model is applied in the context of a single solid–vapor interface, where a planar hydroxide surface is exposed to water vapor at a given relative humidity (RH). The objective is to find the film thickness d_{eq} at which the net disjoining pressure exerted by surface interactions equals the thermodynamic driving force for condensation from the vapor phase.

The total disjoining pressure $\Pi(d)$comprises three contributions:

- van der Waals attraction: modeled using a non-retarded Hamaker expression;
- electrostatic repulsion: based on a constant surface potential approximation with a specified Debye length and ion valence
- hydration repulsion: capturing short-range, exponential decay forces due to structuring of water near the interface.

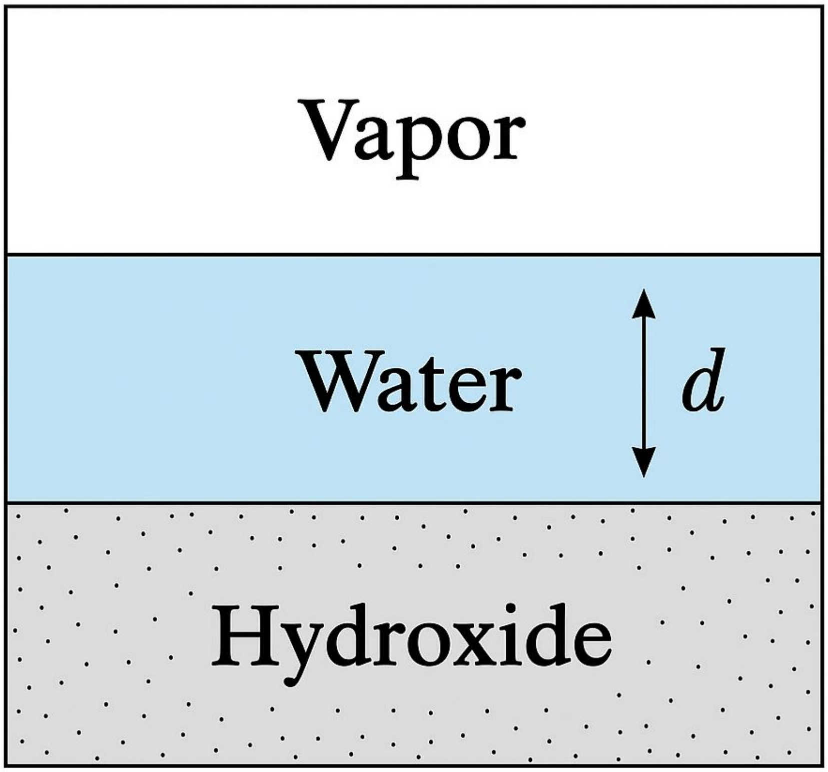

Figure S8 Schematic cross section of model hydroxide surface with physiosorbed water layer

At equilibrium, the net disjoining pressure balances the Kelvin pressure, which reflects the undersaturation of the ambient vapor relative to bulk water. For a single surface exposed to vapor, only one interface contributes to the disjoining pressure, in contrast to symmetric two-surface configurations. Therefore, the force balance condition that defines d_{eq} is

$$\Pi\left(d_{\mathrm{eq}}\right) = \frac{1}{2} \cdot \frac{RT}{V_m} \ln(RH)$$

where R is the gas constant, T is temperature, V_m is the molar volume of water, and RH is the relative humidity. The value of ½ is due to consideration of a single surface.

This equation is solved numerically for d_{eq} using parameter values appropriate for each hydroxide–water system, including Hamaker constants, ionic strength, Debye lengths, and hydration force parameters. The result is a physically grounded estimate of water film thickness as a function of interfacial interactions and ambient humidity, tailored for single-surface geometries.

The total disjoining pressure Π(d) for a thin water film on a single hydroxide surface combines van der Waals, electrostatic, and hydration contributions:

$$\Pi(d) = \Pi_{\mathrm{vdW}}(d) + \Pi_{\mathrm{el}}(d) + \Pi_{\mathrm{hydr}}(d)$$

The van der Waals pressure is:

$$\Pi_{\mathrm{vdW}}(d) = -\frac{A_H}{6\pi d^3}$$

Where:

A_H is the Hamaker constant (J)

d is the film thickness (m)

The electrostatic double layer pressure is:

$$\Pi_{\mathrm{el}}(d) = 64C_0k_BT\left(\tanh\left(\frac{z\gamma}{4}\right)\right)^2 \exp\left(-\frac{d}{\lambda_D}\right)$$

Where:

k_B is the Boltzmann constant (1.38×10^{-23} J/K)

T is the absolute temperature (K)

C_0 is the ion concentration (mol/m^3)

Z is the ionic valence (here 2)

γ = surface potential paramter (dimensionless) $\gamma = \tanh(ez\psi_0/4k_BT)$ and assuming a moderate surface potential ψ_0= 25 mV, gives γ=0.374

λ_D is the Debye length (m), given by:

$$\lambda_D = \sqrt{\frac{\varepsilon\, k_B T}{2z^2 e^2 N_A C_0}}$$

Where:

z is the valence of the ion (e.g., z=2 for Mg^{2+} or Ca^{2+})

e is the elementary charge (1.602×10^{-19} C)

N_A is Avogradro's number (6.022×10^{23} mol^{-1})

$\varepsilon = \varepsilon_0\varepsilon_r$ is the permittivity of water, for H2O ε_r= 78.2 and ε_0=8.854 x 10^{-12} F/m

The hydration pressure is:

$$\Pi_{\mathrm{hydr}}(d) = P_0 \exp\left(-\frac{d}{\lambda_{\mathrm{hydr}}}\right)$$

Where:

P_{0r} is the hydration pressure prefactor (J/m^2)

λ_{hydr} is the hydration decay length (m)

d is the film thickness (m)

The equilibrium film thickness d_{eq} is defined where the total disjoining pressure equals the chemical potential difference induced by vapor undersaturation as given by the equation for $\Pi(d_{eq})$.

Isotope Effect on Film Thickness — H_2O vs. D_2O

The calculated equilibrium film thicknesses show a consistent trend: for both $Mg(OH)_2$ and $Ca(OH)_2$ surfaces, films formed by D_2O are slightly thinner than those formed by H_2O under identical environmental conditions (relative humidity, temperature, and ionic strength).

Dielectric Properties

Heavy water (D_2O) exhibits a lower static dielectric constant than light water (H_2O), approximately 74.6 vs. 78.2 at 25 °C. A lower dielectric constant reduces the electrostatic screening efficiency, thereby enhancing the effective electrostatic interactions between the charged hydroxide surface and surrounding ions. In the context of the DLVO theory, this manifests as a stronger attractive electrostatic double-layer contribution, which promotes thinner equilibrium films.

Hydration Structure and Repulsion

The hydrogen-bond network in D_2O is more structured and less dynamic than in H_2O due to the higher mass of deuterium. This results in stiffer hydration shells around ions and surfaces, which can weaken or shorten the range of hydration repulsion. In force terms, the exponential decay of hydration pressure becomes more abrupt, allowing the film to stabilize at a smaller thickness where attractive and repulsive forces balance.

van der Waals Interaction

Although the Hamaker constant for D_2O is slightly lower than that of H_2O, leading to marginally weaker van der Waals attraction, this effect is relatively minor. The net outcome is still dominated by changes in electrostatic and hydration interactions.

Net Effect

In the combined DLVO + hydration model, these physical differences result in a small but systematic reduction in the equilibrium thickness of D_2O films relative to H_2O films.

Table S5 Film thickness calculations paramters and film thicknesses

System	A_H (J)	C_0 (mol/m^3)	Debye Length (nm)	Effective Surface Potential, γ^2	Hydration Force Prefactor (J/m^2)	Hydration Decay Length (nm)	d_{eq} (nm) RH=70 %	d_{eq} (nm) RH=80 %	d_{eq} (nm) RH=90 %
$Mg(OH)_2$ H_2O	1.20E-20	0.009	50.7	0.14	0.01	0.25	0.30	0.35	0.44
$Mg(OH)_2$ D_2O	1.00E-20	0.009	50.7	0.14	0.01	0.25	0.28	0.33	0.42
$Ca(OH)_2$ H_2O	1.80E-20	20	1.08	0.14	0.01	0.25	0.34	0.40	0.51
$Ca(OH)_2$ D_2O	1.50E-20	20	1.08	0.14	0.01	0.25	0.32	0.37	0.48

Water-Film from Background Variation

Using the background increase for CH1 and MH1 with the following assumptions, we calculate the H_2O film thickness

- Assume spherical particles with a mean diameter as determined by SANS analysis
- Assume H_2O film at liquid density
- Compare H content before and after film formation of given thickness
- Assume background change commensurate with volume of film

Table S6 Calculated water film derived from change in background

	**CH1_N2_H2O**	**MH1_N2_H2O**
Observed bkdg change	1.014	1.061
Calculated H increase	1.017	1.071
Derived thickness	0.1 nm	0.5 nm

These values are somewhat smaller than the 1.3 nm thickness measured by SANS for D_2O on MH4. Previous PM-IRRAS data [1] had shown evidence of a water film for both CH and MH samples, but it grows slowly. See **Figure S6** from that publication which shows the film growth for 10 hours of exposure in humidified N_2.

Mass Balance Equations for Reaction Products

The reaction conditions here are such that the metal ion content is fixed and the volatile components can enter and leave the reacting volume. We note that for samples CH1_CO2_H2O and MH1_CO2_H2O the background increases with reaction. Hence the products contain more hydrogen than reactants. A study of the literature[4, 5] for carbonation reactions near room temperatures suggests that for calcium hydroxide, a highly likely hydrogenous product is amorphous calcium carbonate. Under humidity and temperature conditions such as ours, the water content of this phase is 1.58 moles of H_2O [6]. We write the following equation and calculate hydrogen content change per unit volume

$$Ca(OH)_2 + CO_2 = CaCO_3{\bullet}1.58H_2O + CO_2 + H_2O$$

2H→ 3.16 H (i.e. 15.3 % reaction at 1000 min = 2.18)

This corresponds to 8.9 % H increase and the corresponding background increase at 1000 min is 9.8 %, a reasonable agreement.

For magnesium, the literature suggests the products are likely either amorphous magnesium carbonate or nesquehonite [7-9]. Writing an equation for the reported AMC composition, we obtain

$$Mg(OH)_2 + CO_2 = MgCO_3{\bullet}2.0H_2O + CO_2 + H_2O$$

2H → 4.0 H (i.e. 12.8% reaction at 1000 min = 2.26)

This corresponds to 13% H increase and the corresponding background increase at 1000 min is 12.6%, a reasonable agreement. Nesquehonite, $MgCO_3 \bullet 3H_2O$, would yield a much higher hydrogen content, incompatible with the observed background change and our WANS diffraction patterns.

For MH4_CO2_D2O, the background decreases with reaction, an effect we associate with deuterium exchange during reaction. Assuming the product is AMC, we can estimate the exchange amount.

$Mg(OH)_2 + CO_2 = MgCO_3\bullet(1.34D_2O+0.66H_2O) +CO_2 +H_2O +D_2O$

2H → 0.66 H (i.e. 12.6 % reaction at 1000 min = 1.83)

This corresponds to an 8.5 % H decrease, and the corresponding background change is 5.5 % decrease at 1000 min.

Using these equations, along with extent of reaction from Bragg peak analysis (**Figure 5**) we can analyze the background change to determine the reaction products. In **Figure 6** we summarize the time dependence of the reaction product content and the corresponding background change. This time dependence allows us to calculate the sample composition at any time interval. For SANS scattering models, this composition information is utilized to calculate the neutron scattering length density at individual times and then utilized in the modelling program (**Table S9**).

Interpolation Equations for Background Variation and Bragg Peak Intensity

Table S7 Interpolation of background changes utilized in **Figure 4**

CH1_H2O	
CH1_N2_H2O	Y=1.01E-05x + 1.42E-01
CH1_H2O_CO2	Y=1.44E05x + 1.44E-01
MH1_H2O	
MH1_N2_H2O	Y=3.93E-05x +1.29E-01
MH1_H2O_CO2	Y=1.69E-05x + 1.32E-01
MH4_D2O	
MH4_N2_D2O	Y=-2.31E05x + 1.26E-01
MH4_D2O_CO2	Y=-6.49E06x + 1.21E-01

- Where x is exposure time in minutes and output, Y, is in units of 1/cm

Table S8 Interpolation of Bragg Peak changes utilized in **Figure 4**

CH1-H2O	
CH1_H2O_CO2	Y= -2.60E-04x + 1.70E+00
MH1-H2O	
MH1_H2O_CO2	Y= -2.53E-04x + 1.97E+00
MH4-D2O	
MH4_D2O_CO2	Y= -2.13E-04x + 1.69E+00

- Where x is exposure time in minutes and output, Y, is Bragg peak intensity in arbitrary units

SANS Results

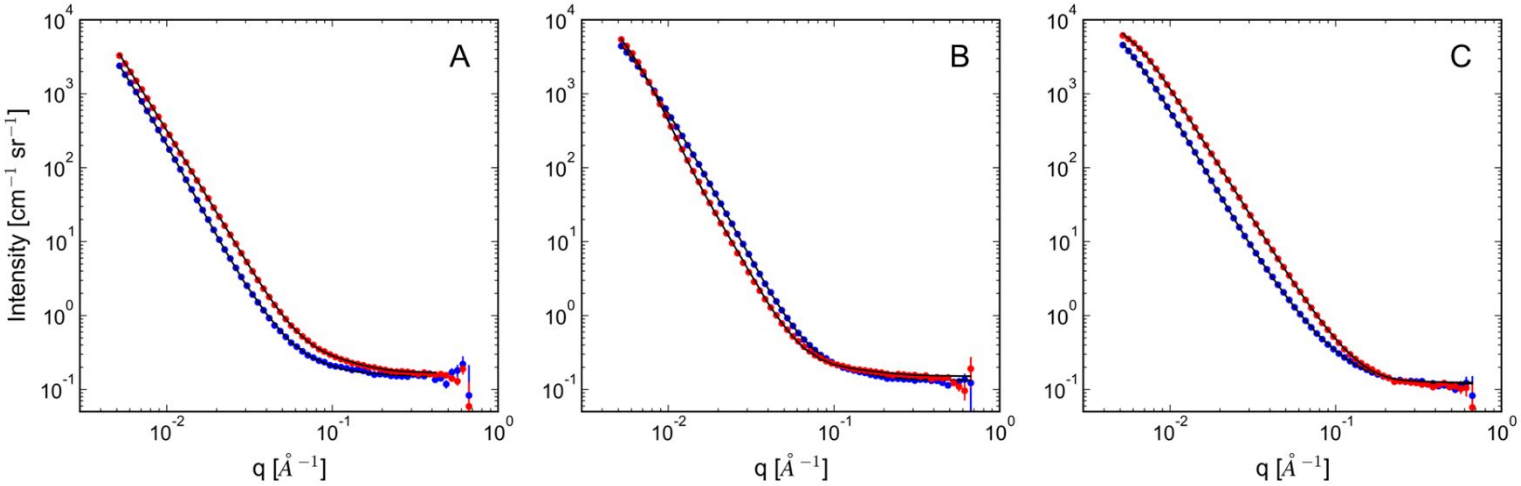

Figure S9 SANS data before (blue) and after (red) carbonation under humidified CO_2.(**A**) CH1_CO2_H2O: increased intensity with carbonation from increases in both meso surface area and diameter, (**B**) MH1_CO2_H2O: decreased intensity with carbonation from decreasing meso-scale and fractal surface areas, and (**C**)MH4_CO2_D2O: significant intensity increase across wide q range from partial deuteration of AMC product with corresponding large increase in contrast. See **Figures 7-10** for structural parameter variation and **Tables S9-S14** for fit parameters.

CH1_CO2_H2O

Table S9 Results $Ca(OH)_2$ with humidified CO_2 Panels are as follows: 1) Summary of reaction conditions, 2) POP1= nano-scale particles, 3) POP2= surface fractal and 4) POP3= meso-scale particles. See discussion of hierarchical scattering model in main text for details on structural components and derived quantities: i.e. Calculated surface area, S_{sf}, R_{eff}

**CH1_CO2_H2O**			
**Sample**	**Time, min**	**React-Frac**	**Contrast Factor**
			(1e28 m-4)

**CH1_CO2_H2O_t1&t2_before t1_1_rev3**	**0.0**	**0.0000**	2.515
**CH1_CO2_H2O_t1_5_rev3**	**193.4**	**0.0296**	2.653
**CH1_CO2_H2O_t1_14_rev3**	**399.9**	**0.0612**	2.796
**CH1_CO2_H2O_t2_23_rev3**	**607.6**	**0.0929**	2.942
**t2_32**	**813.8**	**0.1245**	3.085
**t2_43**	**1065.8**	**0.1630**	3.264

**POP 1:**	**Nano-Scale**		
Aspect ratio	Vol. Frac.	Dmean	Calc. Surf. Area
		(nm)	(m2/cm3)
0.1	0.102	3.73	164.39
0.1	0.113	3.73	182.10
0.1	0.080	4.74	102.22
0.1	0.126	3.73	202.98
0.1	0.127	3.73	204.54
0.1	0.126	3.73	202.65

**POP 2:**	**Surface-Fractal**				
Assumed Rc	So	Ds	Xs	Ssf	Reff
(nm)	(m2/cm3)		(nm)	(m2/cm3)	(m2/cm3)
1.0	3.86	2.200	123.13	10.097	3.573
1.0	9.14	2.196	133.81	23.876	8.913
1.0	9.25	2.101	153.84	15.357	6.042
1.0	9.70	2.143	123.75	19.303	7.991
1.0	8.56	2.153	182.19	18.989	8.243
1.0	8.20	2.206	161.71	23.335	10.717

**POP 3:**	**Meso-Scale**		
Aspect ratio	Vol. Frac.	Dmean	Calc. Surf. Area

		(nm)	(m2/cm3)
1.0	0.090	111.66	8.854
1.0	0.037	126.86	4.174
1.0	0.067	197.15	33.422
1.0	0.063	242.95	45.195
1.0	0.067	242.07	51.132
1.0	0.063	258.36	36.899

MH1_CO2_H2O

Table S10 Results $Mg(OH)_2$ with H_2O humidified CO_2 Panels are as follows: 1) Summary of reaction conditions, 2) POP1= nano-scale particles, 3) POP2= surface fractal , 4) POP3= meso-scale particles, and 5) POP4= Xtra, large particles characteristic of $Mg(OH)_2$ sample. See discussion of hierarchical scattering model in main text for details on structural components and derived quantities: i.e. Calculated surface area, S_{sf}, R_{eff}

**Sample**	**Time, min**	**React-Frac**	**Contrast Factor**
			(1e28 m-4)

**MH1 74733_rev4**	**0.0**	**0.0000**	5.272
**74751**	**208.7**	**0.0268**	5.356
**74769_rev4**	**413.5**	**0.0531**	5.437
**74787_rev4**	**619.0**	**0.0795**	5.522
**74803_rev4**	**802.6**	**0.1031**	5.594
**74821**	**1008.0**	**0.1295**	5.675
**74829**	**1098.5**	**0.1411**	5.712
**74833**	**1143.7**	**0.1469**	5.731

**POP 1:**	**Nano-Scale**		
Aspect ratio	Vol. Frac.	Dmean	Calc. Surf. Area
		(nm)	(m2/cm3)
0.1	0.048	3.23	89.22
0.1	0.047	3.73	76.01
0.1	0.060	3.23	110.93
0.1	0.059	3.03	117.82
0.1	0.056	3.03	111.03
0.1	0.053	3.03	104.39

0.1	0.074	2.73	162.48
0.1	0.073	2.73	159.83

**POP 2:**	**Surface-Fractal**				
Assumed Rc	So	Ds	Xs	Ssf	Reff
(nm)	(m2/cm3)		(nm)	(m2/cm3)	(m2/cm3)
1.0	3.28	2.226	100.00	9.294	5.927
1.0	0.47	2.145	54.82	0.838	0.543
1.0	0.58	2.008	43.12	0.598	0.393
1.0	0.04	2.100	99.98	0.059	0.039
NA					
NA					
NA					
NA					

POP 3:	Meso-Scale		
Aspect ratio	Vol. Frac.	Dmean	Calc. Surf. Area
		(nm)	(m2/cm3)
1.0	0.104	82.05	89.220
1.0	0.127	85.00	76.015
1.0	0.120	84.87	110.925
1.0	0.124	91.26	117.824
1.0	0.132	92.32	111.034
1.0	0.123	90.45	104.395
1.0	0.121	89.78	162.484
1.0	0.119	89.61	159.826

POP4:	Xtra		
Aspect ratio	Vol. Frac.	Dmean	Calc. Surf. Area
		(nm)	(m2/cm3)
4.0	0.0010	166.76	0.014
4.0	0.0377	159.90	0.550

4.0	0.0352	160.19	0.513
4.0	0.0393	160.00	0.573
4.0	0.0072	160.00	0.104
4.0	0.0048	160.00	0.070
4.0	0.0005	160.00	0.007
4.0	0.0001	160.00	0.002

MH4_CO2_D2O

Table S11 Results $Mg(OH)_2$ with D_2O humidified CO_2 Panels are as follows: 1) Summary of reaction conditions, 2) POP1= nano-scale particles, 3) POP2= surface fractal , 4) POP3= meso-scale particles 5) POP4= Xtra, large particles characteristic of $Mg(OH)_2$ sample and 6) POP5= water film. See discussion of hierarchical scattering model in main text for details on structural components and derived quantities: i.e. Calculated surface area, S_{sf}, R_{eff}

**Sample**	**Time, min**	**React-Frac**	**Contrast Factor**
			(1e28 m-4)

**MH4_CO2_D2O**			
**t1_1_74858_vary_ves**	**0.0**	**0.0000**	6.260
**74876_vary_ves**	**201.4**	**0.0254**	6.992
**74894_vary_ves**	**398.1**	**0.0502**	7.725
**74912_vary_ves**	**595.7**	**0.0751**	8.457
**74932_vary_ves**	**814.0**	**0.1026**	9.277
**74934_vary_ves**	**835.8**	**0.1053**	9.336

**POP 1:**	**Nano-Scale**		
Aspect ratio	Vol. Frac.	Dmean	Calc. Surf. Area
		(nm)	(m2/cm3)
0.1	0.0148	3.23	27.58
0.1	0.0005	3.23	0.93
0.1	0.0003	3.23	0.50
0.1	0.0001	3.23	0.13
0.1	0.0000	3.23	0.05
0.1	0.0000	3.23	0.01

**POP 2:**	**Surface-Fractal**				
Assumed Rc	So	Ds	Xs	Ssf	Reff
(nm)	(m2/cm3)		(nm)	(m2/cm3)	(m2/cm3)
1.0	5.78	2.486	22.97	26.540	4.672
1.0	15.12	2.543	22.06	80.997	15.928
1.0	21.12	2.196	17.35	36.888	8.014
1.0	24.19	2.138	17.44	35.872	8.532
1.0	26.21	2.088	16.14	33.463	8.731
1.0	26.30	2.061	15.72	31.118	8.170

**POP 3:**	**Meso-Scale**		

Aspect ratio	Vol. Frac.	Dmean	Calc. Surf. Area
		(nm)	(m2/cm3)
1.0	0.102	97.37	14.044
1.0	0.084	94.87	10.345
1.0	0.057	77.33	5.977
1.0	0.043	71.00	4.498
1.0	0.040	67.28	4.317
1.0	0.041	67.46	4.458

**POP4:**	**Xtra**		
Aspect ratio	Vol. Frac.	Dmean	Calc. Surf. Area
		(nm)	(m2/cm3)
4.0	0.0250	159.68	0.365
4.0	0.0423	159.68	0.617
4.0	0.0250	159.68	0.365
4.0	0.0250	159.68	0.365

4.0	0.0250	159.68	0.365
4.0	0.0250	159.68	0.365

POP4 Constant for this calculation

**POP5:**	**Water-Film**
Film Thickness	Vol. Frac.
(nm)	
1.092	0.0054
1.345	0.0039
1.723	0.0046
1.846	0.0049
1.523	0.0063
1.933	0.0055

MH4_N2_D2O: D_2O film

Using a five component model nano-scale, meso-scale, Xtra, and surface fractal, plus water film scattering, we obtain the values in (**Figure S9 and Table S12)**.

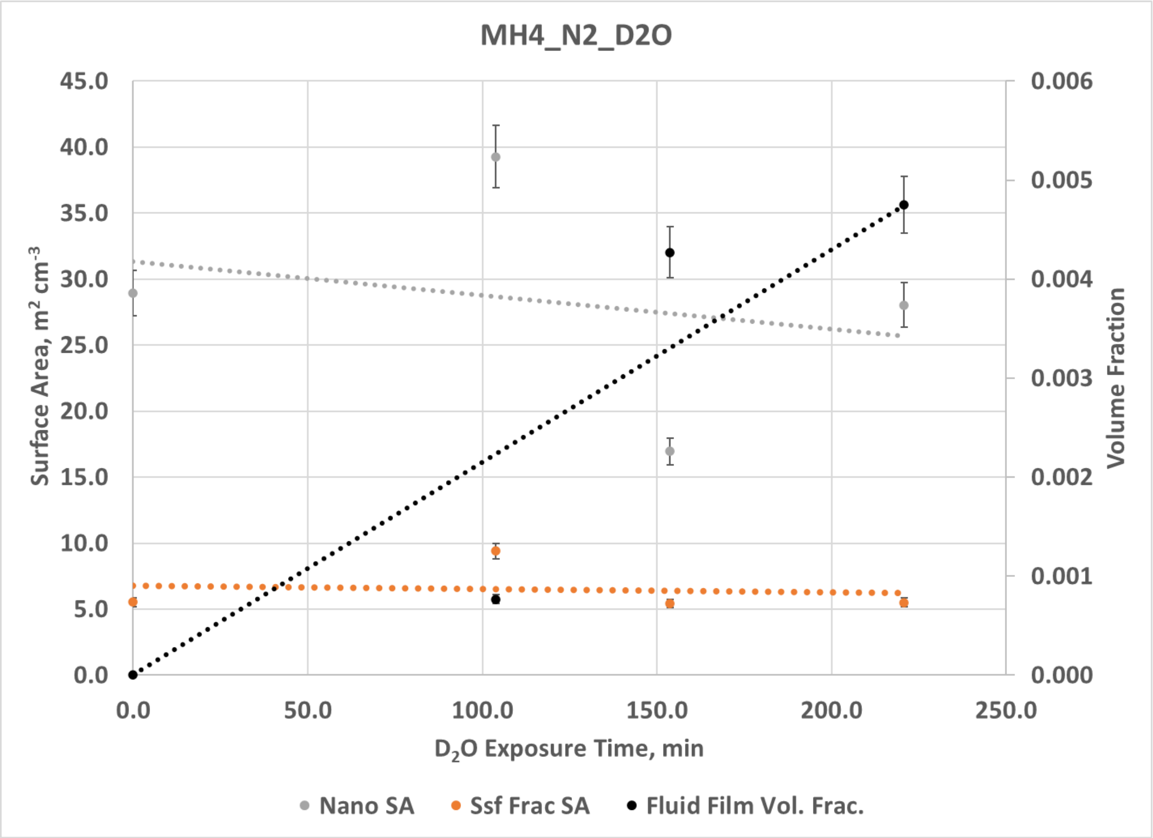

Figure S10. Structural evolution of the MH sample during H–D exchange and D_2O film growth. The major structural change is creation of a fluid film, as shown here from increasing volume fraction. In the main text, **Figure 10**, we show the corresponding change in thickness, reaching a maximum of 1.3 nm in the time interval shown here (**Table S12**). The linear dash lines are guides to the eye, and error bars represent ±6%.

In summary, the majority of the change in scattering arises from D_2O film formation. The evolution of the background signal ("Background" curve in **Figure 3**) provides insight into the extent of deuterium exchange, indicating a composition of 0.96 $Mg(OH)_2$ and 0.04 $Mg(OD)_2$ at *t* = 221 minutes. The "Nano SA" data show a 15% decrease in nano-scale surface area, contrasting to the exponential decay during carbonation (**Figure 9**).

Table S12 Results $Mg(OH)_2$ with D_2O vapor, water film growth. Panels are as follows: 1) Summary of reaction conditions, 2) POP1= nanoscale particles, 3) POP2= surface fractal , 4) POP3= mesoscale particles 5) POP4= Xtra, large particles characteristic of $Mg(OH)_2$ sample and 6) POP5= water film. See discussion of hierarchical scattering model in main text for details on structural components and derived quantities: i.e. Calculated surface area, S_{sf}, R_{eff}

**Sample**	**Time, min**	**React-Frac**	**Contrast Factor**
			(1e28 m-4)
**MH2_N2_D2O_74838 t=0**	**0.0**	**0.0000**	5.270

**74846_vary_SF**	**103.9**	**0.0190**	5.647
**74850_vary_SF**	**153.8**	**0.0280**	6.156
**74856_vary_SF**	**220.8**	**0.0400**	6.260

**POP 1:**	**Nano-Scale**		
Aspect ratio	Vol. Frac.	Dmean	Calc. Surf. Area
		(nm)	(m2/cm3)
0.1	0.020	4.23	28.93
0.1	0.028	4.23	39.26
0.1	0.012	4.23	16.95
0.1	0.020	4.23	28.03

**POP 2:**	**Surface-Fractal**			
Assumed Rc	So	Ds	Xs	Ssf
(nm)	(m2/cm3)		(nm)	(m2/cm3)
1.0	2.25	2.229	50.98	5.538

1.0	3.82	2.229	50.98	9.397
1.0	2.20	2.229	50.98	5.419
1.0	2.25	2.230	48.76	5.511

**POP 3:**	**Meso-Scale**		
Aspect ratio	Vol. Frac.	Dmean	Calc. Surf. Area
		(nm)	(m2/cm3)
1.0	0.104	107.38	17.636
1.0	0.093	101.53	16.466
1.0	0.100	102.41	17.243
1.0	0.104	107.71	17.639

**POP4:**	**Xtra**		
Aspect ratio	Vol. Frac.	Dmean	Calc. Surf. Area
		(nm)	(m2/cm3)

4.0	0.0250	159.68	0.365
4.0	0.0250	159.68	0.365
4.0	0.0250	159.68	0.365
4.0	0.0250	159.68	0.365

POP4 constant for these calculations

**POP5:**	**Water-Film**
Film Thickness	Vol. Frac.
(nm)	
0.000	0.0000
0.821	0.0008
0.802	0.0043
1.302	0.0048

CH1_CO2_D2O: D_2O film

For CH1_CO2_D2O we exposed an already partially carbonated (12%) sample to first a purge gas of dry N_2 then to a CO_2-D_2O mixture. WANS data does not show any further carbonation over the relatively short 100 minute

exposure. However, as seen in **Figure S4** a D_2O film forms. We apply the fitting routine and obtain the values in **Table S13**, giving a D_2O film with volume fraction 0.004 and thickness 0.5 nm. These are similar to the values obtained for MH4_N2_D2O between 100 min and 150 min. For CH1_N2_H2O and MH1_N2_H2O, we attempted to fit parameters of the H_2O film for data sets, but the vesicle volume fraction would refine to zero when included. The signal to noise was insufficient for this weak scattering.

Table S13 Results $Ca(OH)_2$ with D_2O vapor, water film growth. Panels are as follows: 1) Summary of reaction conditions, 2) POP1= nanoscale particles, 3) POP2= surface fractal , 4) POP3= mesoscale particles and 5) POP5= water film. See discussion of hierarchical scattering model in main text for details on structural components and derived quantities: i.e. Calculated surface area, S_{sf}, R_{eff}

**Sample**	**Time, min**	**React-Frac**	**Contrast Factor**
			(1e28 m-4)
**CH1_N2_74709_rev1**	**0.0**	**0.0000**	3.264
**CH1_D2O_74719_revb**	**111.1**	**0.0000**	3.264

**POP 1:**	**Nano-Scale**		

Aspect ratio	Vol. Frac.	Dmean	Calc. Surf. Area
		(nm)	(m2/cm3)
0.1	0.152	3.73	245.96
0.1	0.121	3.73	195.37

**POP 2:**	**Surface-Fractal**				
Assumed Rc	So	Ds	Xs	Ssf	Reff
(nm)	(m2/cm3)		(nm)	(m2/cm3)	(m2/cm3)
1.0	16.36	2.062	48.82	20.852	1.969
1.0	13.43	2.129	120.72	24.962	2.357

**POP 3:**	**Meso-Scale**		
Aspect ratio	Vol. Frac.	Dmean	Calc. Surf. Area

		(nm)	(m2/cm3)
1.0	0.033	402.01	48.908
1.0	0.077	1052.40	19.413

**POP5:**	**Water-Film**
Film Thickness	Vol. Frac.
(nm)	
0	0
0.600	0.0035

MH1_CO2_H2O: H_2O film

Table S14 Results $Mg(OH)_2$ with H_2O vapor, water film growth. Panels are as follows: 1) Summary of reaction conditions, 2) POP1= nanoscale particles, 3) POP2= surface fractal , 4) POP3= mesoscale particles 5) POP4= Xtra, large particles characteristic of $Mg(OH)_2$ sample and 6) POP5= water film. See discussion of hierarchical scattering model in main text for details on structural components and derived quantities: i.e. Calculated surface area, S_{sf}, R_{eff}

**Sample**	**Time, min**	**React-Frac**	**Contrast Factor**
			(1e28 m-4)
**MH1 74733_vary_ves**	**0.0**	**0.0000**	5.272
**74803_vary_ves**	**802.6**	**0.1031**	5.594
**74829_vary-ves**	**1098.5**	**0.1411**	5.712

**POP 1:**	**Nano-Scale**		
Aspect ratio	Vol. Frac.	Dmean	Calc. Surf. Area
		(nm)	(m2/cm3)
0.1	0.056	3.23	103.78
0.1	0.053	3.03	105.89
0.1	0.070	2.73	154.15

**POP 2:**	**Surface-Fractal**			
Assumed Rc	So	Ds	Xs	Ssf

(nm)	(m2/cm3<br>)		(nm)	(m2/cm3<br>)
1.0	3.25	2.15<br>3	501.2<br>7	8.435
NA				
NA				

**POP 3:**	**Meso-Scale**		
Aspect ratio	Vol. Frac.	Dmean	Calc. Surf. Area
		(nm)	(m2/cm3)
1.0	0.111	90.19	19.359
1.0	0.122	90.17	11.592
1.0	0.111	87.86	10.137

**POP4:**	**Xtra**		
Aspect ratio	Vol. Frac.	Dmean	Calc. Surf. Area
		(nm)	(m2/cm3)

4.0	0.0250	159.68	0.365
4.0	0.0250	159.68	0.365
4.0	0.0250	159.68	0.365

POP 4 constant for these calculations

**POP5:**	**Water-Film**
Film Thickness	Vol. Frac.
(nm)	
1.000	5.00E-03
1.000	5.00E-05
1.000	1.25E-05

PM-IRRAS: Carbonation

PM-IRRAS investigations of $Mg(OH)_2$ and $Ca(OH)_2$ in D_2O humidified CO_2 align with our observations reported previously for experiments in H_2O.[1] Peak positions for hydrated magnesium carbonate (1447 cm^{-1} and 1510 cm^{-1}) appear in slightly shifted positions from the H_2O study (by roughly 20 cm^{-1}) but show consistent behavior with exposure time (**Figure S11B**). The spectra also show signatures of a D_2O water film from peaks at 1216 cm^{-1} and 2512 cm^{-1} (inverted) that diminish under N_2 purge. These results are consistent with our study of $Mg(OH)_2$ dosed with H_2O -humidified CO_2. The $Ca(OH)_2$ sample shows the growth of an amorphous calcium carbonate (ACC) phase and a D_2O film layer. Shifting backgrounds during high humidity dosing convolute trends, evidenced by sharp discontinuities in peak profiles upon atmosphere change to N_2 (**Figure S11D**) for 1524 cm^{-1} and 1410 cm^{-1} peaks.[10] The peaks corresponding to D_2O at 1208 cm^{-1} and 2524 cm^{-1} show a more gradual change that indicates removal of the D2O water layer upon N_2 purge, though the magnitude of change is also convoluted by the baseline shift. The hydroxide O-H peak at 3654 cm^{-1} changes in intensity, but no distinct hydroxide O-D peak is apparent, indicating there is not an exchange reaction taking place in $Ca(OH)_2$.

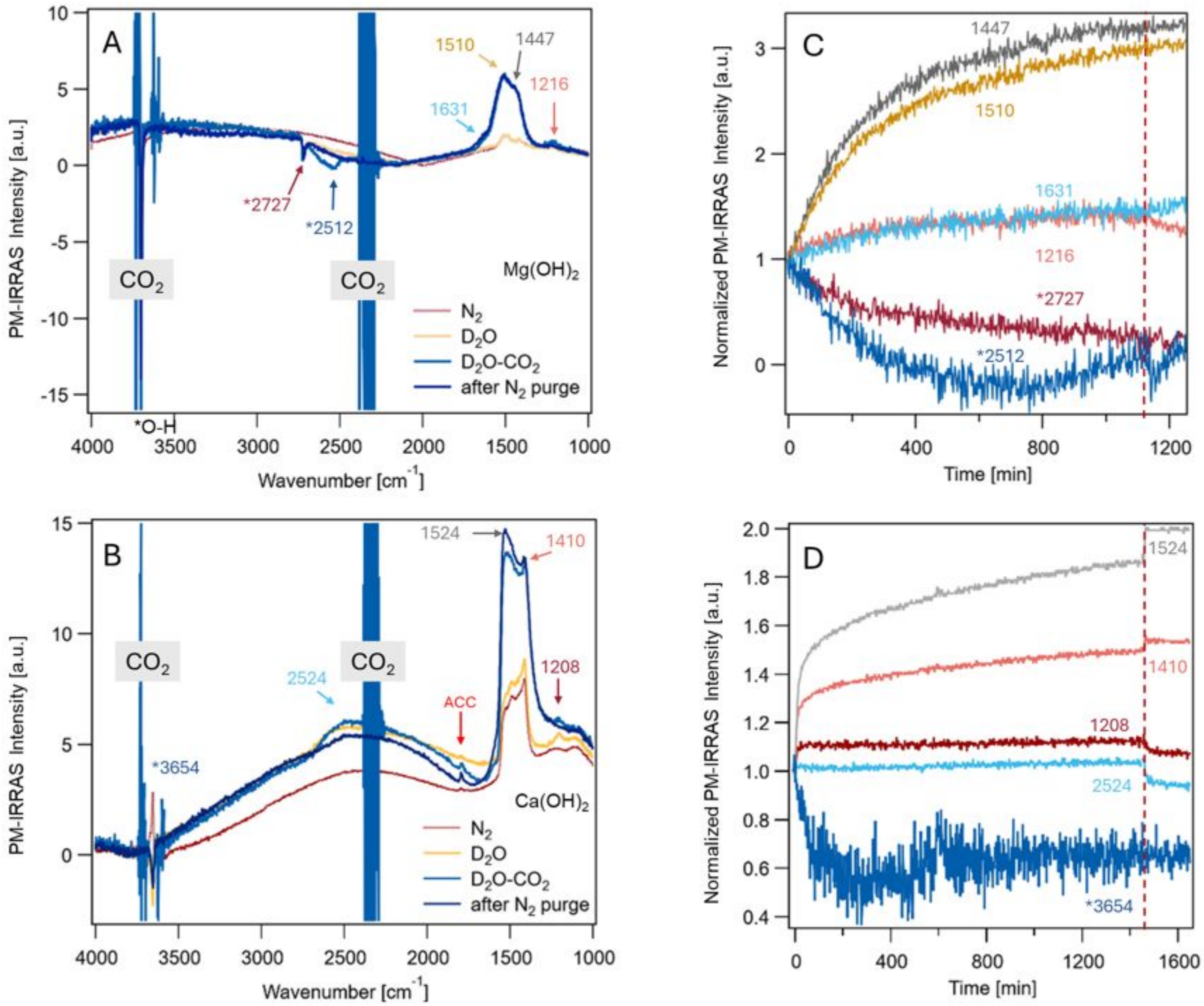

Figure S11. PM-IRRAS spectra for different dosing conditions of (**A**) $Mg(OH)_2$ and (**B**) $Ca(OH)_2$, showing vibrational features labeled by peak positions that are tracked as a function of time in panels (**C**) and (**D**), respectively. Formation of amorphous calcium carbonate (ACC) is identified in $Ca(OH)_2$. The vertical dashed lines in (**C, D**) mark the transition from

D_2O-humidified CO_2 exposure to N_2 purge. Results here closely follow those obtained in similar experiments for H_2O. (See King [1])

References

(1) King, H.; Murphy, R.; Baumann, A.; Allen, A.; Nguyen, H. G. T.; DeBeer-Schmitt, L.; Ilavsky, J. Carbonation of Alkaline Earth Metal Hydroxides: Structure across Nano-to Mesoscales. *Energy & Fuels* **2025**, *39* (10), 4866–4879.
(2) SASVIEW. *SASVIEW*. https://www.sasview.org/, 2025. (accessed).
(3) Ilavsky, J.; Jemian, P. R. Irena: tool suite for modeling and analysis of small-angle scattering. *Journal of Applied Crystallography* **2009**, *42* (2), 347–353.
(4) Fernandez-Martinez, A.; Kalkan, B.; Clark, S. M.; Waychunas, G. A. Pressure-induced polyamorphism and formation of 'aragonitic'amorphous calcium carbonate. *Angewandte Chemie* **2013**, *125* (32), 8512–8515.
(5) Rodriguez-Navarro, C.; Kudłacz, K.; Cizer, Ö.; Ruiz-Agudo, E. Formation of amorphous calcium carbonate and its transformation into mesostructured calcite. *CrystEngComm* **2015**, *17* (1), 58–72.
(6) Radha, A.; Forbes, T. Z.; Killian, C. E.; Gilbert, P.; Navrotsky, A. Transformation and crystallization energetics of synthetic and biogenic amorphous calcium carbonate. *Proceedings of the National Academy of Sciences* **2010**, *107* (38), 16438–16443.
(7) Tanaka, J. Y.; Kawano, J.; Nagai, T.; Teng, H. Transformation process of amorphous magnesium carbonate in aqueous solution. *Journal of Mineralogical and Petrological Sciences* **2019**, *114* (2), 105–109.
(8) Yamamoto, G.-i.; Kyono, A.; Okada, S. Temperature dependence of amorphous magnesium carbonate structure studied by PDF and XAFS analyses. *Scientific Reports* **2021**, *11* (1), 22876.
(9) Yang, J.; Han, Y.; Luo, J.; Leifer, K.; Strømme, M.; Welch, K. Synthesis and characterization of amorphous magnesium carbonate nanoparticles. *Materials Chemistry and Physics* **2019**, *224*, 301–307. DOI: https://doi.org/10.1016/j.matchemphys.2018.12.037.

(10) Takeuchi, M.; Kikuchi, T.; Kondo, A.; Kurosawa, R.; Ryu, J.; Matsuoka, M. Near-Infrared Spectroscopic Analysis─Formation of Ca (OH) 2 and Ca (OD) 2 by Hydration of CaO with H2O and D2O. *The Journal of Physical Chemistry C* **2023**, *127* (13), 6406–6413.
